# Supplementary material for: An item sorting heuristic to derive equivalent parallel test versions from multivariate items
Source: PLoS One. 2023 Apr 25;18(4):e0284768. doi: 10.1371/journal.pone.0284768 (PMC10128941; doi:10.1371/journal.pone.0284768)
Supplement: S1 File — Normierung eines Benenn- und semantischen Entscheidungstests für biologische und manipulierbare Objekte in deutscher Sprache. (PDF) [file pone.0284768.s001.pdf]

Normierung eines Benenn- und semantischen Entscheidungstests für biologische und manipulierbare Objekte in deutscher Sprache

Masterarbeit von Eva-Maria Berger

Eingereicht bei PD Dr. Klemens Gutbrod

Abteilung Allgemeine Psychologie und Neuropsychologie

Institut für Psychologie, Universität Bern

Juni 2012

Korrespondenzadresse:
Eva Berger
[redacted]

Abstract

Wortfindungsstörungen kommen bei diversen neurologischen Erkrankungen, unfallbedingten Verletzungen und altersbedingten Degenerationsprozessen vor, welche die linke, sprachdominante Hirnhemisphäre betreffen. Ziel dieser Arbeit war die Normierung und die Konstruktion von Benenn- und semantischen Entscheidungstests für biologische und manipulierbare Objekte zur Diagnose von Wortfindungsstörungen bei deutschsprachigen Patienten. Die Parameter Wortfrequenz, Naming Agreement, Wortkomplexität, Bildübereinstimmung sowie Kongruenz hatten einen bedeutsamen Einfluss auf die Reaktionszeit für die Bildbenennung und die semantische Entscheidung. Zudem ergaben sich durch die Parameter Wortlänge, Naming Agreement, Bildübereinstimmung und Vertrautheit unterschiedliche Einflüsse auf die biologischen und manipulierbaren Objekte. Es wurden ein Benenntest mit zwei Paralleltestversionen à 58 Items und ein semantischer Entscheidungstests mit zwei Paralleltestversionen à 116 Items konstruiert, welche für alle bedeutsam erwiesenen Parameter und die Objektklasse kontrolliert wurden.

Normierung eines Benenn- und semantischen Entscheidungstests für biologische und manipulierbare Objekte in deutscher Sprache

Wortfindungsstörungen kommen bei diversen neurologischen Erkrankungen, unfallbedingten Verletzungen und altersbedingten Degenerationsprozessen vor, welche die linke, häufig sprachdominante Hirnhemisphäre betreffen. Wortfindungsstörungen äussern sich in einer mangelnden Verfügbarkeit bzw. reduzierten Abrufbarkeit des intendierten Wortes. Dadurch kommt es zu einem sprachlichen Suchverhalten, welches sich in unterschiedlichsten Formen manifestieren kann, wie „lange Pausen, die mit Interjektionen ausgefüllt werden; Ausweichen in inhaltsarme Redefloskeln; perseveratorische Wiederholung von gerade gebrauchten Wörtern; Satzabbrüche und Fortführen des Themas in variierter Form; Ausweichen in Pantomime, Gestik und Mimik“ (Weniger, 2006, S.357f).

Die meisten Personen haben mit diesem Phänomen eigene Erfahrungen aus ihrem Alltag, so kann beispielsweise das gesuchte Ziel-Wort trotz grosser Anstrengung nicht abgerufen werden. Dieses Phänomen wird umgangssprachlich mit „das Wort liegt mir auf der Zunge“ umschrieben (engl.: Tip-of-the-tongue-Phenomenom (TOT); Brown & McNeill, 1966; Levelt, 1989). Solche alltäglichen Wortfindungsprobleme bei sprachgesunden Personen unterscheiden sich jedoch hinsichtlich ihrer Qualität und Quantität massiv von klinisch bedeutsamen Wortfindungsstörungen.

Wortfindungsstörungen treten am häufigsten als Symptom bei aphasischen Patienten auf. Aphasien definiert man als erworbene Sprachstörungen, die als Folge einer Erkrankung des zentralen Nervensystems (ZNS) auftreten (Huber & Ziegler, 2009). Im Erwachsenenalter sind die häufigsten medizinischen Ursachen Schlaganfälle und im Kindes- sowie Jungendalter Hirnverletzungen durch Unfälle. Aphasien sind als multimodale und supramodale Sprachstörungen zu definieren (Huber, Poeck & Weniger, 2002). Sie werden

linguistisch als Schädigungen in den verschiedenen Bereichen des Sprachsystems beschrieben, wobei das Lexikon, die Syntax, die Phonologie und die Semantik[1] betroffen sein können. Die Störungen zeigen sich zudem in allen expressiven und rezeptiven sprachlichen Verarbeitungsmodalitäten. Das heisst beim Sprechen, Verstehen, Lesen und Schreiben, wobei prinzipiell bei all diesen Modalitäten dieselben linguistischen Beeinträchtigungsmerkmale nachweisbar sind (Huber et al., 2002). Grundsätzlich werden vier grosse Standardsyndrome mit jeweils charakteristischen Leitsymptomen unterschieden: die globale Aphasie, die Wernicke-Aphasie, die Broca-Aphasie und die amnestische Aphasie. Wortfindungsstörungen stellen das Leitsymptom der amnestischen Aphasie dar, können jedoch auch bei allen anderen aphasischen Syndromen auftreten.

Sprachverarbeitungsprobleme, wie beispielsweise die Benennleistung, können nicht nur durch neurologische Erkrankungen oder durch unfallbedingte Hirnverletzungen beeinflusst werden, sondern auch durch den normalen kognitiven Alterungsprozess (vgl. Mayr, 2006; Reischies, 2006). Somit wird die Wahrscheinlichkeit für Probleme mit der Wortfindung mit steigendem Alter grösser. Jedoch zeigt sich, dass die Wortfindung insbesondere bei pathologischen Degenerationsprozessen im Vergleich zum normalen kognitiven Alterungsprozess stark beeinträchtigt sein kann.

Das Demenzsyndrom vom Alzheimer-Typ, bei welchem das Erkrankungsalter um das siebte Lebensjahrzehnt liegt, stellt die bekannteste und auch die häufigste demenzielle Erkrankung dar (Poeck & Hartje, 2002). Bei dieser Demenzform ist zunächst eine kortikale Atrophie der bilateralen parietotemporalen und mediobasalen temporalen Regionen festzustellen. Im späteren Verlauf der Erkrankung ist jedoch das gesamte Gehirn von den

---

[1] Lexikon: aktiver und passiver Wortschatz, Lexikon der gelernten Wörter;
Syntax: Theorie der Satzstruktur;
Phonologie: Theorie der Lautstruktur;
Semantik: Bedeutung von Wörtern und Sätzen
(nach De Bleser, 2006)

pathologischen Veränderungen betroffen. Gemäss Reischies (2006) gehören Störungen des episodischen Gedächtnisses zu den Initialsymptomen der Alzheimer-Demenz. Zudem treten Beeinträchtigungen in der Sprache häufig relativ früh im Krankheitsverlauf auf. Hierbei fallen zuerst Wortfindungsstörungen auf, weshalb man dieses Stadion auch als anomische Phase der Alzheimer-Demenz bezeichnet. Hodges, Patterson, Graham und Dawson (1996) vertreten die Annahme, dass es sich bei der anomischen Symptomatik zunächst hauptsächlich um eine Störung des semantischen Systems handelt. Die Autoren begründen diesen Standpunkt dadurch, dass die Syntax und die Spontansprache, mit Ausnahme der Wortfindungsprobleme, zunächst unauffällig sind. Hingegen zeigen sich erhebliche Defizite, wenn Betroffene Wörter einer semantischen Kategorie (bspw. Werkzeuge oder Früchte) finden sollen.

Bei der semantischen Demenz, bei welcher ebenfalls Wortfindungsstörungen auftreten, sind vor allem der Temporalpol und die präfrontalen Strukturen des Gehirns vom degenerativen Prozess betroffen, weshalb dieser Typ zu den frontotemporalen Demenzen (FTD) gehört (Münte, 2009). Hierbei kommt es zu einem fortschreitenden Verlust semantischer Konzepte, sodass die Bedeutung von Wörtern, Gesichtern (Prosopagnosie) und Objekten nicht mehr verstanden wird und aus diesem Grund auch nicht mehr benannt werden kann (Poeck & Hartje, 2002). Dabei bleiben sowohl die Spontansprache, als auch das episodische und autobiographische Gedächtnis lange Zeit gut erhalten. Im weiteren Verlauf werden Objekte nicht mehr an ihren visuellen Merkmalen erkannt, was einer visuellen Agnosie entspricht.

Als weitere Ursache von Wortfindungsstörungen ist die primär progressive Aphasie (PPA) zu nennen. Diesem Typ, welcher zu den frontotemporalen Lobärdegenerationen (FTLD) gehört, liegt häufig ein Nervenzelluntergang in den sprachrelevanten kortikalen Regionen zugrunde (Baumann, Tolnay & Monsch, 2009). Bei der PPA wird ein langsam

fortschreitender Sprachzerfall beobachtet, welcher während mindestens zwei Jahren isoliert vorhanden ist (Mesulam & Weintraub, 1992). Es lassen sich drei verschiedene Formen unterscheiden: die nicht flüssige progressive Aphasie, die flüssige progressive Aphasie und die gemischte progressive Aphasie (siehe Croot, 2009 für einen Überblick). Von diesen Subtypen zeigt nur die flüssige progressive Aphasie Wortfindungsstörungen bei gleichzeitig intakter Sprache und erhaltener Grammatik. Im weiteren Verlauf der Erkrankung weisen viele Betroffene Alltagsschwierigkeiten auf, welche nicht mehr ausschliesslich durch Sprachsymptome erklärt werden können. In diesem Stadium gleichen die Symptome denen einer frontotemporalen Demenz, wobei jedoch die Sprachstörung weiterhin, im Vergleich zu anderen Symptomen, die dominanteste bleibt (Le Rhun, Richard & Pasquier, 2005).

Sprachproduktionsmodell und neuronale Grundlagen der Sprache

Eines der wichtigsten psycholinguistischen Modelle der Sprachproduktion ist das Modell von Levelt (1989). Gemäss Levelt werden für die Produktion von Sprache drei Module – der Konzeptualisierer, der Formulator und der Artikulator – seriell durchlaufen. Auf der Ebene des Konzeptualisierers entsteht eine sprachunabhängige Mitteilungsabsicht bzw. ein Mitteilungskonzept, welches zur weiteren Verarbeitung an den Formulator weitergeleitet wird. Auf der Ebene des Formulators finden zwei sich nicht überlappende Phasen statt, wobei das Lemma und das dazugehörige Lexem zusammengeführt werden. Dabei erfolgt zuerst durch das Lemma ein Zugriff auf alle lexikalischen und grammatikalischen Eigenschaften eines Konzeptes im mentalen Lexikon und danach wird durch das Lexem die phonologische Struktur enkodiert, welche für die phonetische Form eines Wortes benötigt wird. Mit Abschluss der zweiten Phase werden im Artikulator die erhaltenen Informationen in ein artikulomotorisches Programm transformiert, wodurch das Wort artikuliert werden kann.

Gemäss Levelt, Roelofs und Meyer (1999) entstehen Wortfindungsstörungen nicht im Konzeptualisierer, weil die Mitteilungsabsicht einer betroffenen Person durch die Anwendung von Kompensationsstrategien (wie z.B. das Ausweichen auf Pantomime, Gestik und Mimik) deutlich erkennbar ist und auf nonverbaler Ebene ausgedrückt werden kann. Die Störung der Wortproduktion kann auch nicht auf das letzte Modul, den Artikulator, zurückgeführt werden, da die Spontansprache der Betroffenen meistens kaum betroffen ist und lediglich ein bestimmtes Wort nicht abgerufen werden kann. Die Störung des Wortabrufs muss demnach im Formulator entstehen (Levelt et al., 1999). Hierbei erfolgt zwar ein Zugriff auf die lexikalische Eigenschaft des gesuchten Wortes, aber die phonologische Struktur, also das Lexem, kann nicht enkodiert werden. Dadurch steht die phonetische Form des Wortes nicht zur Verfügung, weshalb das Wort nicht artikuliert werden kann. Diverse Studien zum TOT-Phänomen unterstützen diese Theorie, bei welchen die Probanden das Zielwort zwar nicht artikulieren, aber diverse Angaben wie beispielsweise den Anfangsbuchstaben und die Anzahl der Silben (Brown & McNeill, 1966) oder das Geschlecht (Vigliocco, Antonini & Garrett, 1997) zum gesuchten Wort angeben konnten.

Neben theoretischen Sprachmodellen spielen auch die neurologischen Grundlagen der Sprache eine Rolle für das Verständnis von Wortfindungsstörungen. Erste Zuordnungen von Sprachprozessen zu spezifischen Hirnregionen erfolgten durch Paul Broca (1861) und Carl Wernicke (1874) aufgrund der von ihnen beschriebenen Fallstudien. Broca stellte als Sitz der Sprachproduktion die dritte Frontalwindung (Gyrus frontalis inferior) fest und Wernicke beschrieb posteriore Anteile des Gyrus temporalis superior als zentral für das Sprachverständnis. Zudem ging Wernicke davon aus, dass die beiden erwähnten Sprachzentren über Fasern (Fasciculus arcuatus) miteinander verbunden sind. Neuere Studien, welche sich mit der kortikalen Repräsentation und mit der Darstellung sprachrelevanter Regionen befassen, haben gezeigt, dass die beiden Sprachzentren weitaus

grösser und weit mehr Regionen an diesem Prozess beteiligt sind. Ein Überblick der neuronalen Grundlagen für das Sprachverständnis und die Sprachproduktion gibt Price (2010). DeLeon et al. (2007) erläutern zudem die neuronalen Regionen, welche spezifisch an der Bildbenennung beteiligt sind.

Vigneau et al. (2006) unterscheiden aufgrund ihrer Meta-Analyse der sprachlichen Verarbeitungsprozesse zwischen einem ventralen und dorsalen Pfad der semantischen Verarbeitung im Temporalkortex. Der ventrale Verarbeitungspfad umfasst Anteile des Gyrus fusiformis, posteriore Anteile des Gyrus temporalis inferior, den Temporalpol, anteriore Anteile des Gyrus temporalis superior und der Gyrus angularis. Dieser Pfad wird insbesondere bei der konzeptuell-semantischen Verarbeitung von visuellen Stimuli benötigt. Der dorsale Pfad wird hingegen für die lexikalisch-semantische Verarbeitung von auditiven Sprachreizen benötigt und umfasst posteriore Anteile des Gyrus temporalis superior, posteriore Anteile des Temporalkortex, anteriore Anteiles des Gyrus frontalis inferior und Anteile des Gyrus präzentralis. Gemäss Vigneau et al. sind Wortfindungsstörungen weitgehend auf einen Beeinträchtigten dorsalen Verarbeitungspfad zurückzuführen.

Muss eine Wortbedeutung gezielt abgerufen werden oder muss zwischen gleichzeitig aktivierten Wortbedeutungen unterschieden werden, sind insbesondere anteriore Anteile des Gyrus frontalis inferior für die Verarbeitung notwendig (vgl. Badre & Wagner, 2007). Diese Hirnregion ist durch den Fasciculus uncinatus mit anterioren Anteilen des Temporalkortex verbunden. Ist diese Verbindung beeinträchtigt, können solche spezifischen Aufgabenstellungen nicht mehr korrekt gelöst werden.

Klinische Diagnostik von Wortfindungsstörungen

Für die klinische Diagnose von Wortfindungsstörungen werden Benenntests eingesetzt, in welchen die Patienten verschiedene abgebildete Objekte benennen sollen. Die

im Folgenden genannten klinischen Tests stellen keine vollständige Auflistung der bestehenden Diagnoseverfahren dar, sondern sollen einen Überblick über die im deutschsprachigen Raum am meist verwendeten Tests geben.

Der Boston Naming Test (Kaplan, Goodglass & Weintraub, 1983), als wichtigster international etablierter Benenntest, wurde in englischer Sprache entwickelt und dient als Einzeltest einzig dazu die Benennleistung zu messen. Normalerweise wird die Benennleistung eines Patienten nicht mittels eines Einzeltests, sondern innerhalb einer ganzen Testbatterie abgeklärt. Diese werden für die Diagnostik von Aphasien eingesetzt und sind in ihrer Durchführung und Auswertung häufig standardisiert. Solche Testbatterien sind beispielsweise der Minnesota Test for Differential Diagnosis of Aphasia (MTDDA; Schuell, 1965; in deutscher Sprache von Delavier & Graham, 1981), Boston Diagnostic Aphasia Examination (BDAE; Goodglass & Kaplan, 1972), Psycholinguistic Assessment of Language Processing in Aphasia (Kay, Lesser & Coltheart, 1992) und Western Aphasia Battery (WAB; Kertesz, 1982). Für die deutsche Sprache wurden Testbatterien wie der Aachener Aphasie-Test (AAT; Huber, Poeck, Weniger & Willmes, 1983), der LeMo – Lexikon modellorientiert (De Bleser, Cholewa, Stadie & Tabatabaie, 2004) und die Wortproduktionsprüfung (Blanken, Döppler & Schlenk, 1999) entwickelt, wobei der Benenntest jeweils einen Untertest darstellt. Von diesen Testbatterien in deutscher Sprache ist jedoch lediglich der AAT vollständig standardisiert. Des Weiteren sind Bildbatterien zu nennen, welche hauptsächlich in der Forschung verwendet werden. Die Bildbatterie von Snodgrass und Vanderwart (1980), welche 260 schwarz-weisse Strichzeichnung umfasst (in farbiger Version von Rossion & Pourtoise, 2004), stellt die bekannteste dieser Batterien dar.

Mit den heutzutage verwendeten Diagnoseverfahren gehen verschiedene Schwierigkeiten einher, welche die Aussagekraft der Testergebnisse beeinträchtigen kann. Die gängigen Diagnoseverfahren sind dafür konzipiert Wortfindungsstörungen bei

Aphasieerkrankungen zu erfassen. Werden die gleichen Tests für die Erfassung von Wortfindungsstörungen bei anderen Patientengruppen wie beispielsweise Demenzpatienten verwendet, so sind die Tests oftmals zu leicht beziehungsweise zu wenig sensitiv, um die vorhandenen Schwierigkeiten korrekt abbilden zu können. Des Weiteren wird die Beeinträchtigung jeweils nach Anzahl erhaltener Punkten in einem Test festgestellt, jedoch spielt auch die Geschwindigkeit, mit welcher ein Bild benannt werden kann, eine entscheidende Rolle, da auch eine korrekte Bildbenennung, für welche jedoch viel Zeit benötigt wird, eine Beeinträchtigung in der Sprachverarbeitung darstellt. Zudem gibt es in der Regel keine Paralleltestversionen, weshalb Therapiefortschritte oder Verbesserungen in der Wortfindung bzw. der dafür benötigten Zeit beispielsweise nach Operationen nicht spezifisch erfasst werden können.

Effekte von linguistischen Parametern auf die Benennleistung

Die Verwendung von übersetzten klinischen Tests beinhaltet häufig, dass linguistische Unterschiede zwischen den verschiedenen Sprachen ungenügend beachtet werden. Bei der Entwicklung eines Tests wird darauf geachtet, dass in der Endversion unterschiedliche Schwierigkeitsgrade der Testitems vorhanden sind, damit eine gute Differenzierung zwischen Patienten und sprachgesunden Personen möglich ist. Die Schwierigkeitsgrade der Testitems sind abhängig von linguistischen Parametern, welche in den verschiedenen Sprachen stark variieren können. Bates et al. (2003) haben in einer Pionierstudie das Benennen von Bildern in sieben verschiedenen Sprachen hinsichtlich unterschiedlicher linguistischer Parameter miteinander verglichen, wobei sie gleiche und unterschiedliche Einflüsse der linguistischen Parameter auf die verschiedenen Sprachen festgestellt haben. Für die von den Autoren einbezogenen linguistischen Parameter konnten

schon früher Effekte auf die Benennleistung gezeigt werden, wie im Folgenden diskutiert wird.

Durch diverse Studien wurde belegt, dass die Wortfrequenz einen Einfluss auf den lexikalen Abruf hat (bspw. Oldfield & Wingfield, 1965; Jescheniak & Levelt, 1994; Barry, Morrison & Ellis, 1997). Je häufiger ein Wort in der Alltagssprache verwendet wird, desto schneller, beziehungsweise leichter, kann das Wort abgerufen werden. Die Frequenz eines Wortes wird erhoben, indem kalkuliert wird, wie häufig das Zielwort innerhalb eines repräsentativen Korpus von Texten vorkommt. Das Leipziger Wortschatzportal (http://wortschatz.uni-leipzig.de) stellt Wortfrequenzklassen in Deutsch zur Verfügung, bei welchen das Auftreten des Zielwortes mit dem Auftreten des Wortes „der" verglichen wird. Das Vergleichswort „der" stellt in der deutschen Sprache das höchstfrequente Wort dar. Je tiefer die Frequenzklasse eines Wortes, desto häufiger ist es im Textkorpus der Universität Leipzig vorhanden und desto häufiger wird es wahrscheinlich in der Alltagssprache verwendet.

Ausserdem spielt die Namensübereinstimmung auch eine grosse Rolle. Die Namensübereinstimmung (engl.: Naming Agreement; Bates et al., 2003) wird definiert als die Anzahl äquivalenter Begriffe für ein und dasselbe Objekt. Kann ein Objekt mit verschiedenen Begriffen benannt werden, so hat das Objekt ein tiefes Naming Agreement. Wird ein Objekt von allen Personen gleich benannt, beziehungsweise hat das Objekt einen eindeutigen Namen, entspricht dies einem hohen Naming Agreement. Durch diverse Studien wurde erwiesen, dass Bilder von Objekten mit einem hohen Naming Agreement, schneller und genauer benannt werden, als Bilder von Objekten mit einem tiefen Naming Agreement (bspw. Bates et al. 2003; Barry et al., 1997; Genzel, Kerkhoff & Scheffter, 1995).

Die Länge eines Wortes bzw. aus wie vielen Silben ein Wort besteht, hat ebenfalls einen Einfluss auf die Reaktionszeit in der Bildbenennung. Die Reaktion bei längeren

Wörtern ist im Vergleich zu kürzeren Wörtern langsamer (D'Amico, Devescovi & Bates, 2001; Szekely et al., 2005). Des Weiteren wurden in einer Studie von Klapp, Anderson und Berrian (1973) monosilbige Wörter schneller benannt als zweisilbige Wörter. Diese Effekte auf die Reaktionszeit sind jedoch eher klein und verschwinden, wenn verschiedene Konfundierungsfaktoren kontrolliert werden. Gemäss Bates et al. (2003) stellt einer dieser Faktoren das Zipfsche Gesetz (Zipf, 1965) dar, welches besagt, dass zwischen der Länge eines Wortes und seiner Auftretenshäufigkeit eine negative Korrelation besteht. Das heisst, häufige, hochfrequente Wörter sind generell kürzer als seltene, tieffrequente Wörter. Daraus lässt sich schlussfolgern, dass die Komplexität eines Wortes auch einen Einfluss auf die Latenzzeit der Bildbenennung haben kann. Komplexe Wörter werden definiert als zusammengesetzte Wörter (Nomina Composita) oder als Flexionsformen eines Wortes. Demnach sollten komplexe Wörter im Vergleich zu einfachen Wörtern länger und tieffrequenter sein.

In diversen Studien (vgl. bspw. Bates, Devescovi, Pizzamiglio, D'Amico & Hernandez, 1995) wurde festgestellt, dass ein Frikativ zu Beginn eines Wortes die Reaktionszeit für die Bildbenennung beeinflusst. Frikative sind Reibelaute wie f, s, v und z. Diese Laute werden von Mikrophonen häufig ungenügend registriert, weswegen die Messung der Reaktionszeit verspätet ausgelöst wird und dies somit fälschlicherweise zu einer längeren Latenzzeit führt.

Aufgrund der diskutierten linguistischen Parameter wird die Problematik der Verwendung von übersetzten Tests ersichtlich. Die Anordnung der Items in einem Test ist für die Originalsprache sinnvoll, da diese anhand der erfassten linguistischen Parameter erfolgte. Dieselbe Reihenfolge in Übersetzungen ist jedoch nicht unbedingt sinnvoll, da sich die Sprachen hinsichtlich linguistischer Merkmale deutlich unterscheiden können. Beispielsweise würde das englische Wort „desk“ ein einfaches Item darstellen, da es aus wenigen

Buchstaben besteht, einsilbig ist, nicht mit einem Frikativ beginnt und eine hohe Frequenz[2] aufweist. Dasselbe Wort in deutscher Sprache, „Schreibtisch", würde hingegen zu den schwierigeren Items zählen, da es aus vielen Buchstaben besteht, mehrsilbig ist, ein komplexes Nomen darstellt, mit einem Frikativ beginnt und eine niedrige Frequenz[3] aufweist. Daraus lässt sich schlussfolgern, dass für ein korrektes Testergebnis entweder übersetzte Tests zwingend hinsichtlich der linguistischen Parameter der „neuen" Sprache angepasst oder die Tests nur in ihrer ursprünglichen Sprache verwendet werden sollten.

Kategorienspezifische Effekte auf die Benennleistung

Neben den linguistischen Parametern können auch Wortkategorien die Benennleistung unterschiedlich beeinflussen. Aphasiker und sprachgesunde Personen benannten in einer Studie von Mätzig, Druks, Masterson und Vigliocco (2009) Objektbilder (Nomen) generell schneller und häufiger korrekt als Tätigkeitsbilder (Verben). Die Autoren erklärten diese Ergebnisse damit, dass die Benennung von Tätigkeitsbildern mit einer aufwendigeren und komplexeren Verarbeitung dieser Wortkategorie zusammenhängt: Im Gegensatz zu Nomen werden Verben später erworben (bspw. Bates et al., 1994), weisen eine komplexere grammatikalische Struktur auf (bspw. Saffran, Schwartz & Marin, 1980), haben eine komplexere semantische Repräsentation (bspw. Vinson & Vigliocco, 2002) und lassen sich durch Bilder schlechter darstellen (Bird, Howard & Franklin, 2000). Des Weiteren wurde in manchen Studien eine doppelte Dissoziation zwischen Broca- und Wernicke-Aphasikern in der Produktion von Nomen und Verben festgestellt (bspw. Zingeser & Berndt, 1990). Dabei erbringen viele nicht fluente Broca-Aphasiker bessere Leistungen bei Objektbenennungen im Gegensatz zu Tätigkeitsbenennungen. Das umgekehrte Bild zeigt sich

[2] log ln-korrigierte Frequenz nach CELEX: 4.52 (siehe Bates et al., 2003): Je höher der korrigierte logarithmische Wert eines Wortes, desto höher die Wortfrequenz.
[3] Häufigkeitsklasse: 13; „der" ist ca. 2^{13} mal häufiger als das gesuchte Wort.

bei fluenten Wernicke-Aphasikern, welche Tätigkeiten wesentlich besser benennen können als Objekte.

Verschiedene Objektklassen können die Benennleistung ebenfalls unterschiedlich beeinflussen. Viele Patienten, welche eine Schädigung in den linkshemisphärischen Gebieten des anterioren, medialen und inferiorenTemporallappens erlitten haben, können belebte, biologische Objekte (z.B. Tiere, Pflanzen) schlechter benennen, währendem die Benennung von unbelebten, angefertigten bzw. manipulierbaren Objekten (z.B. Möbel, Werkzeuge) nicht beeinträchtigt ist (Woollams, Cooper-Pye, Hodges & Patterson, 2008). Liegt eine Schädigung der linksseitigen frontoparientalen Hirnareale vor, so ist das umgekehrte Muster ersichtlich (Martin & Chao, 2001). Chao, Haxby und Martin (1999) konnten zudem bei Aufgaben mit Tierstimuli erhöhte Aktivierungen im lateralen Teil des Gyrus fusiformis hervorrufen, wohingegen Werkzeuge bilateral den mittleren Teil des Gyrus fusiformis stärker aktivierten. Eine Erklärung für diese Beeinträchtigung wird auf eine unterschiedliche Gewichtung der Merkmale zur Identifizierung der Objektklassen zurückgeführt (Woollams et al., 2008). Belebte Objekte werden vor allem durch sensorisch erfassbare Merkmale wie Farbe, Form und Grösse unterschieden. Viele Exemplare einer semantischen Gruppe können sich hierbei eine Vielzahl von semantischen Merkmalen teilen. Beispielsweise können Tiere wie Hund, Katze, Marder, Wolf oder Zebra alle mit den Merkmalen „vier Beine, ein Fell und ein Schwanz“ beschrieben werden. Eine exakte Differenzierung der einzelnen Exemplare ist dementsprechend schwieriger, da viele ähnliche Exemplare ausgeschlossen werden müssen und ist daher auch mit einer längeren Verarbeitungszeit verbunden. Unbelebte bzw. manipulierbare Objekte werden hingegen aufgrund ihrer Funktion differenziert. Chao und Martin (2000) stellten eine erhöhte Aktivierung des linken ventralen Teils des prämotorischen Cortex bei der visuellen Verarbeitung von Werkzeugen im Vergleich zu

Tieren, Gesichtern und Häusern fest, was sie der Assoziation verschiedener Handbewegungen zuschreiben. Dieses Gebiet wird bereits bei der Vorstellung einer Handbewegung, wie sie beispielsweise mit einem Werkzeug durchgeführt wird, aktiviert.

Gellatly und Gregg (1975) liessen sprachgesunde Personen entscheiden, ob semantisch ähnliche oder unähnliche Bild-Wort-Paare kongruent oder inkongruent waren. Im Vergleich zu semantisch unähnlichen Paaren ergaben sich signifikant längere Reaktionszeiten als für die Entscheidung bei semantisch ähnlichen Bild-Wort-Paaren. Zudem wurde für die Entscheidung bei inkongruenten Wortpaaren mehr Zeit benötigt als bei kongruenten Paaren. Die Autoren erklären diesen Unterschied im Entscheidungsprozess durch einen längeren Verarbeitungsprozess bei semantischen ähnlichen Paaren. Um solche Entscheidungen treffen zu können, werden jeweils zwei Merkmale der Stimuli miteinander verglichen, wobei das Resultat, ob die Merkmale übereinstimmen oder nicht für die definitive Entscheidung bereitgehalten wird. Die definitive Entscheidung über die Kongruenz bzw. Inkongruenz der Stimuli wird getroffen, wenn eine individuell festgelegte Anzahl an Vergleichsresultaten erreicht wird. Bei semantisch unähnlichen Paaren benötigt es für die Entscheidung nur wenige Vergleichsresultate, da davon ausgegangen werden kann, dass auch wenige übereinstimmende Merkmale der Stimuli sehr unwahrscheinlich sind. Bei semantisch ähnlichen Paaren benötigt es hingegen eine Vielzahl an Merkmalsvergleichen, weil aufgrund der semantischen Ähnlichkeit wenige übereinstimmende Merkmale sehr wahrscheinlich sind und somit noch nicht genügend Informationen für eine Entscheidung über die Kongruenz vorliegen würden.

Effekte von Bildparametern auf die Benennleistung

Die Darstellungsart der Bilder kann ebenfalls Effekte auf die Benennleistung haben. Für die prototypische Darstellung der Objekte werden in den meisten Benenntests schwarz-

weisse Strichzeichnungen verwendet. Wird jedoch die unterschiedliche Verarbeitung von belebten und unbelebten Objekten in Betracht gezogen, müsste davon ausgegangen werden, dass farbige Bilder die Prototypikalität von belebten Objekten positiv unterstützten würde, da belebte Objekte häufig eine einzige typische Farbe haben (beispielsweise eine gelbe Banane, eine rote Erdbeere) und somit eine schnellere Identifizierung erfolgen könnte. Price und Humphreys (1989) bestätigten diese Annahme in ihrer Studie, in welcher sich zeigte, dass farbige Bilder die Benennung von Objekten im Gegensatz zu schwarz-weissen Strichzeichnungen vereinfachte, wobei sich die Farbe insbesondere auf belebte Objekte positiv auswirkte. Gleichermassen stellten Rossion und Pourtois (2004) durch die Verwendung von farbigen Bildern höhere Bilderbenennungen und ebenfalls kürzere Reaktionszeiten fest. Für unbelebte Objekte erfolgt jedoch nicht immer eine Steigerung der Prototypikalität, weil verschiedene Personen eine andere Farbe als prototypisch dafürhalten können (bspw. eine weisse, rote, grüne oder zweifarbige Tasse). Ausserdem könnte bei Patienten, welche zusätzlich Beeinträchtigungen in der Farberkennung aufweisen, die Benennleistung zusätzliche gemindert werden, wenn farbige Bilder präsentiert werden. Oder es könnte sogar dazu führen, dass ein Objekt nicht aufgrund seiner Prototypikalität, sondern aufgrund der Farbe erkannt wird. Durch die Verwendung von farbigen Bildern wäre somit die Vergleichbarkeit der Benennleistung von Patienten mit verschiedenen Ätiologien nicht gewährleistet.

Ein weiterer Effekt zeigt sich in der Vertrautheit eines Objekts. Die Vertrautheit wird definiert, als wie wahrscheinlich es für eine Person ist, mit einem Konzept bzw. Objekt in Kontakt zu treten oder darüber nachzudenken. Snodgrass und Vanderwart (1980) sowie auch Genzel et al. (1995) haben festgestellt, dass eine kürzere Reaktionszeit durch eine höhere Vertrautheit mit dem Konzept zustande kommt. Des Weiteren zeigen Patienten mit

semantischen Prozessstörungen bessere lexikalische Abrufleistungen bei hoch vertrauten Objekten als bei weniger vertrauten Objekten (bsp. Woollams et al., 2008).

Die visuelle Komplexität kann die Benennleistung ebenfalls beeinflussen. Die visuelle Komplexität eines Bildes bezieht sich auf die Detailanzahl, mit welcher ein Bild gezeichnet ist. Gemäss Ellis und Morrison (1998) sowie auch Genzel et al. (1995) beeinflusst die visuelle Komplexität die Reaktionszeit, sodass eine hohe visuelle Komplexität mit höheren Reaktionszeiten verbunden ist. Ausserdem korreliert die visuelle Komplexität negativ mit der Vertrautheit (Snodgrass & Vanderwart, 1980; Genzel et al., 1995). Demnach geht eine hohe Vertrautheit mit visuell weniger komplexen Bildern einher.

Des Weiteren ist die Bildübereinstimmung zu nennen, welche ebenfalls einen Effekt auf die Latenzzeit bei der Bildbenennung haben kann. Gemäss Snodgrass und Vanderwart (1980) ist die Bildübereinstimmung (engl. Image Agreement) definiert, als wie gut das Bild mit der eigenen Vorstellung des Konzepts übereinstimmt. Gemäss den Autoren gehen hohe Bildübereinstimmungen mit kürzeren Reaktionszeiten einher, da das Objekt durch die höhere Übereinstimmung schneller erkannt wird.

Ziel der vorliegenden Studie

Ziel der vorliegenden Arbeit ist die Normierung einer Bilderauswahl über ihre linguistischen, kategorienspezifischen und bildspezifischen Parameter bei nicht hirnverletzten Personen. Hierfür werden eine Benenn-, eine Entscheidungs- und eine Beurteilungsaufgabe durchgeführt. Anhand der gewonnenen Daten erfolgt die Erstellung eines Benenn- und eines semantischen Entscheidungstest mit jeweils zwei Paralleltestversionen, welche über alle erfassten Parameter kontrolliert sind.

In die Untersuchung werden in Anlehnung an die Studie von Snodgrass und Vanderwart (1980) und Bates et al. (2003) die Parameter Wortlänge (Silben und Buchstaben),

Wortfrequenz, Naming Agreement, Wortkomplexität, Initialfrikativ, Bildübereinstimmung, Vertrautheit, visuelle Komplexität, die Reaktionszeit und die Korrektheit (Anzahl korrekter Antworten, engl.: Accuracy) fürs Benennen von dargebotenen Bilder miteinbezogen. Ebenfalls werden die Objektklasse und spezifisch für die semantische Entscheidungsaufgabe die Kongruenz als weitere dichotome Parameter eingefügt, da deren Einfluss auf die Reaktionszeit aus früheren Studien bekannt ist (bspw. Woollams et al., 2008; Gellatly & Gregg, 1975).

Für die vorliegende Studie wird bewusst auf farbige Bilder verzichtet und es werden keine Bilder von Tätigkeiten verwendet. Es werden prototypische schwarz-weisse Strichzeichnungen verwendet, um zu gewährleisten, dass die Bilder nur aufgrund der Prototypikalität identifiziert werden können und die Benennleistung über verschiedene Patientengruppen vergleichbar bleibt. Bilder von Tätigkeiten werden des Weiteren nicht miteinbezogen, da bei einer vorgängigen Arbeit (Hoffmann, 2010) Tätigkeiten, welche als statische Bilder dargestellt wurden, nicht vergleichbar eindeutig und schnell benennbar waren wie Bilder von Nomen. Als Ersatz für Tätigkeitsbilder werden unbelebte Nomina verwendet, welche manipulierbar sind und somit mit einer Tätigkeit assoziiert werden können.

Anhand dieser in der vorliegenden Arbeit erstellten Benenn- und semantischen Entscheidungstests wird das Ziel angestrebt künftig Wortfindungsstörungen über verschiedene Patientengruppen und Beeinträchtigungsgrade hinweg reliabel erfassen zu können.

Methode

Versuchspersonen

An der Studie nahmen insgesamt 52 Personen im Alter von 22 bis 81 Jahren teil, davon 27 Männer und 25 Frauen, sowie 49 Rechts- und 3 Linkshänder. Das durchschnittliche

Alter lag bei 42,79 Jahren (SD = 21,17). Die Teilnehmer verfügten durchschnittlich über 14,12 Bildungsjahre (Schuljahre und Berufsausbildungsjahre; SD = 2,55). Voraussetzung für die Teilnahme an der Studie war Deutsch bzw. Schweizerdeutsch als Muttersprache und keine bisherigen Erkrankungen in den Bereichen der Neurologie oder Psychiatrie zu haben. Die Rekrutierung der Teilnehmer erfolgte innerhalb des Bekanntenkreises der Autorin und anhand einer Ausschreibung an der Senioren-Universität Bern.

Stimuli

In der Studie wurden insgesamt 128 Wörter verwendet, welche auf 64 Wortpaare aufgeteilt werden können (siehe Tabelle 1). Hiervon bestehen 32 Wortpaare aus biologischen Objekten. Die Wortpaare der biologischen Objekte wurden so ausgewählt, dass sich die Wörter eines Paares entweder aufgrund ihrer Semantik (bspw.: Huhn - Gans: beides Federvieh auf dem Bauernhof) und/oder ihrer Phonologie (bspw.: Eule – Ente: beides zweisilbige Wörter, beide beginnen mit einem „E“ und enden mit einem „e“) ähnlich sind oder starke Assoziationen mit einem bestimmten Konzept zwischen ihnen bestehen (bspw.: Krokodil – Elefant: beides Tiere von Afrika). Die restlichen 32 Wortpaare bestehen aus manipulierbaren Objekten. Das Kriterium für diese Wortpaare war, dass die Objekte mit den Händen manipuliert werden können und die jeweilige Manipulation eines Wortpaares mit der gleichen bzw. ähnlichen Tätigkeit ausgeführt werden kann (bspw. Tätigkeit/Verb zum Wortpaar Pinzette - Zange: rausziehen). Das zu den Wörtern passende Bildmaterial wurde von verschiedenen Quellen ausgewählt. 24 Bilder stammen aus dem Bildkorpus von Snodgrass und Vanderwart (1980). 76 weitere Strichzeichnungen wurden aus dem Bildarchiv[4] von Dr. Dorothea Weniger zur Verfügung gestellt. Für weitere 28 Wörter

[4] Die aus dem Bildarchiv von Dr. Dorothea Weniger verwendeten prototypischen Strichzeichnungen wurden erstellt für experimentelle Untersuchungen zur Sprachverarbeitung bei Aphasie von Dr. Dorothea Weniger und Prof. Dr. Walter Huber an der Neurologischen Klinik des Universitätsklinikum und für Prof. Dr. Rudolf Cohen im Rahmen eines Sonderforschungsprojekts der DFG.

konnten keine passenden Bilder gefunden werden, weshalb Vorlagen aus dem Internet entnommen wurden, aus welchen eine Mitarbeiterin des Inselspitals in Bern neue Strichzeichnungen erstellte. Alle verwendeten Bilder wurden zusätzlich mit einem Bildbearbeitungsprogramm (Photoshop CS3 10.0®; Adobe®) überarbeitet, sodass die Darstellungsform der einzelnen schwarz-weissen Strichzeichnungen aus den verschiedenen Bildquellen keine grossen Abweichungen mehr vorwies.

Material

Die Datenerhebung für die Benenn[5]- und Entscheidungsaufgabe wurde auf einem Littlebit Laptop (Betriebssystem Windows XP) mit dem Computerprogramm E-Prime® 2.0 (© 2007 Psychology Software Tools, Inc.) durchgeführt. Um während der Benennaufgabe die exakten Reaktionszeiten erfassen zu können, war eine Button-Box (Serial Response Box; © 1996-2003 Psychology Software Tools, Inc.; Modell # 200A) mit einem Kopfbügelmikrophon (Typ HS1-P Headset; Røde Microphones) über einen seriellen Anschluss, welcher mit einem Adapter (Typ USB2.0 to Serial Adapter; LogiLink) auf einen USB-Anschluss konvertiert wurde, am Laptop angeschlossen. Zusätzlich wurde direkt am Laptop ein Ansteckmikrophon (Typ RP-VC201E-S; Panasonic) angeschlossen, über welches die mündlichen Antworten der Studienteilnehmer parallel zur Reaktionszeitmessung digital aufgezeichnet und als WAV-Dateien auf dem Laptop abgespeichert wurden. Zur Protokollierung der Antworten und möglichen fehlerhaften Reaktionszeiten während der Benennaufgabe wurde ein Protokollbogen in Papierform verwendet. Für die fehlerhaften Reaktionszeiten wurde im Nachhinein die korrekte Reaktionszeit mit dem Computerprogramm WavePad Sound Editor 5.08 (NCH Software) ermittelt. Die

[5] Die Programmierung mit E-Prime ® 2.0 der Benennaufgabe wurde von der vorangegangenen Masterarbeit von Hoffmann (2010) übernommen.

Datenerhebung der Beurteilungsaufgabe wurde in Papierform durchgeführt, wofür ein 13-seitiges Dokument (A4 Format) erstellt wurde, auf welchem die 128 Bilder aufgeführt waren.

Design

Die Untersuchung wurde mit allen Versuchspersonen in der gleichen Reihenfolge durchgeführt. Zuerst wurden die 128 Bilder benannt, danach musste bei 256 Bild-Wort-Paaren eine Entscheidung getroffen werden und zuletzt wurden die 128 Bilder beurteilt. Die Bilder waren in jedem Teil des Experiments fixiert randomisiert angeordnet, um zu vermeiden, dass Bilder des gleichen Wortpaares direkt hintereinander erscheinen. Es gab keine Kontrollgruppe.

Durchführung

Den Versuchspersonen wurde zu Beginn des Experiments eine kurze mündliche Erklärung über das Ziel des Experiments gegeben. Es wurde darauf hingewiesen, dass sie eine Normierungsstichprobe darstellen und dass anhand ihrer Reaktionszeiten festgelegt werden soll, wie lange gesunde, nicht hirnverletzte Personen im Durchschnitt benötigen, um Bilder zu benennen und Bild-Wortpaare zu beurteilen. Zusätzlich soll die Qualität der Bilder festgestellt werden.

Benennaufgabe

Den Versuchspersonen wurde mit einer schriftlichen Erklärung auf dem Bildschirm erklärt, dass sie Bilder sehen werden, welche sie ganz spontan und ohne lange zu überlegen in hochdeutscher Sprache mit dem ersten Wort, welches ihnen einfällt, benennen sollten. Sie wurden darauf hingewiesen, Verzögerungslaute wie „öhm“ oder „ähm“ vor der eigentlichen Antwort zu vermeiden, damit eine korrekte Reaktionszeit erfasst werden kann. Damit sich die Teilnehmer an den Versuchsablauf gewöhnen konnten, wurden 5 Beispielaufgaben

(geometrische Figuren: Quadrat, Kreis, Dreieck, Stern, Kreuz) vor der eigentlichen Testdurchführung gelöst. Danach wurden die 128 Bilder benannt, wobei es jeweils nach 32 Bildern eine Pause gab, welche mit einem beliebigen Tastendruck beendet werden konnte.

Die Bilder wurden mit einer Auflösung von 200x200 Pixel in der Mitte des Bildschirms präsentiert. Die Trials begannen mit der Darstellung eines Fixationskreuzes für 3000ms. Danach wurde das Bild zentriert in der Mitte des Bildschirms präsentiert, wobei die Reaktionszeit beim ersten Geräusch, welches die Response Box wahrgenommen hatte, gemessen wurde. Im linken oberen Ecken des Bildschirms erschien ein kleines Kreuz, sobald eine Reaktionszeit gemessen wurde. Dadurch konnte die Versuchsleiterin überprüfen, ob die Messung erfolgreich war oder ob die Messung aufgrund eines Geräuschs zu früh erfolgte bzw. keine Reaktionszeit gemessen wurde. Diese Angaben wurden entsprechend auf einem Protokollblatt vermerkt, um später gegebenenfalls eine Korrektur der Reaktionszeit vornehmen zu können. Nachdem die Versuchsperson das Bild benannt hatte, wurde durch einen beliebigen Tastendruck der nächste Trial ausgelöst.

Semantische Entscheidungsaufgabe

Anhand einer schriftlichen Instruktion auf dem Bildschirm wurde erklärt, dass jeweils gleichzeitig ein Bild und ein Wort auf dem Bildschirm erscheinen werden und die Aufgabe darin besteht zu beurteilen, ob das Bild mit dem Wort übereinstimmt oder nicht. Hierbei sollten die Versuchspersonen nicht lange nachdenken, sondern ganz spontan eine Entscheidung treffen. Ein kongruentes Bild-Wort-Paar stellte beispielsweise ein Bild einer Ente und dem Schriftzug „Ente“ dar. Ein inkongruentes Bild-Wort-Paar wurde analog zu den erstellten Wortpaaren kombiniert, indem das Bild das erste und der Schriftzug das zweite Wort eines Wortpaares darstellte. Zur Eingewöhnung an den Versuchsablauf durchliefen die Versuchspersonen vor der eigentlichen Durchführung 5 Beispielaufgaben mit kongruenten und inkongruenten Bild-Wort-Paaren anhand geometrischer Figuren.

Danach wurden die 256 Bild-Wort-Paare (128 kongruente und 128 inkongruente Paare) in randomisierter Weise präsentiert. Dabei wurde jeweils gleichzeitig ein Bild (200 x 200 Pixel) in der oberen Hälfte und ein Wort (Schriftgrösse 30) in der unteren Hälfte des Bildschirms zentriert dargestellt. Die Trials begannen mit der Präsentation eines Fixationskreuzes für 3000ms. Danach erschien das Bild-Wort-Paar solange bis die Versuchsperson ihre Entscheidung mittels eines Tastendrucks getroffen hatte. Wenn das Bild-Wort-Paar kongruent war, so sollte die rechte Pfeiltaste (→), bzw. bei einer Inkongruenz die linke Pfeiltaste (←) gedrückt werden.

Beurteilung der Bilder

Zuletzt bestand die Aufgabe darin, die Bilder in einem 13-seitigen Dokument nochmals schriftlich zu benennen und anhand dreier Parameter (Bildübereinstimmung, Vertrautheit und die visuelle Komplexität) auf jeweils einer 5-stufigen Likertskala (von *1= nicht ...* bis *5 = ganz ...*) zu beurteilen. Die Instruktion, welche von den Studien von Snodgrass und Vanderwart (1980) sowie Genzel et al. (1995) übernommen wurden, erhielten die Versuchspersonen schriftlich auf einem weiteren Blatt. Die Teilnehmer wurden instruiert, nochmals jedes Bild so schnell und eindeutig wie möglich mit dem ersten Namen, der ihnen einfällt auf Schriftdeutsch zu benennen. Für die Beurteilung der Bildübereinstimmung erhielten die Versuchspersonen die Instruktion, ihre eigene Vorstellung des Objekts mit dem Bild zu vergleichen und zu entscheiden, wie stark diese beiden übereinstimmen. Die visuelle Vertrautheit wurde definiert als wie wahrscheinlich die Versuchspersonen mit dem Konzept/Objekt in Kontakt treten oder darüber nachdenken, das heisst, die Versuchspersonen sollten beurteilen, wie vertraut/alltäglich beziehungsweise unvertraut/fremd die abgebildeten Objekte bzw. Konzepte für sie sind. Die visuelle Komplexität war definiert als „Detailanzahl und Anzahl der Striche aus denen das Bild besteht“. Dies bedeutet, je grösser die Anzahl der Details und Striche, desto visuell komplexer ist das Bild. Zur besseren Einschätzung der

beiden Parameter visuelle Vertrautheit und Komplexität wurden Beispielitems vorgelegt, welche extremen Ausprägungen der beiden Parameter entsprachen (hohe visuelle Komplexität: Motorrad; hohe Vertrautheit: menschliche Hand; niedrige visuelle Komplexität: Richtungspfeil; niedrige Vertrautheit: Gepard). Nachdem die Versuchspersonen alle Bilder beurteilt hatten, war die Durchführung beendet und die Teilnehmer wurden verabschiedet.

Resultate

Es wurde das Ziel verfolgt, zwei Parallelversionen für einen Benenntest und zwei Parallelversionen für einen semantischen Entscheidungstest zu erstellen. Die Paralleltestversionen sollten jeweils gleich viele biologische wie manipulierbare Objekte beziehungsweise Items enthalten und sich bezüglich der erhobenen Parameter nicht unterscheiden. Im Folgenden wird zuerst die Itemanalyse für beide Tests gemeinsam beschrieben und danach die weiteren Auswertungen für den Benenn- und den semantischen Entscheidungstest getrennt erläutert. Beim Benenntest wurden für die Analyse der erfassten Parameter Korrelationen und Mann-Whitney *U*-Tests und für die Testkonstruktion der Wilcoxon- bzw. McNemar X^2-Test verwendet. Die Analyse des semantischen Entscheidungstests erfolgte für die Parameter ebenfalls mit Korrelationen und Mann-Whitney *U*-Tests sowie zusätzlich anhand einer zweifaktoriellen Varianzanalyse mit Messwiederholung.

Itemanalyse

Um die Qualität der Items zu prüfen, wurde eine Itemanalyse durchgeführt, durch welche die qualitativ hochstehenden bzw. ungenügenden Items identifiziert werden können. Hierfür wurde jeweils für den Benenn- und den semantischen Entscheidungstest für jedes Item ein Schwierigkeitsindex berechnet, welcher angibt, wie gross der Anteil von Probanden

ist, die ein Item ‚richtig' beantwortet haben (Michel & Conrad, 1982 zit. nach Fisseni, 1997). Bei Items mit einer dichotomen Kodierung (richtig/falsch) wird der Schwierigkeitsindex berechnet, indem die Anzahl der Richtiglöser einer Stichprobe durch die Anzahl der Gesamtstichprobe dividiert wird. Der Schwierigkeitsindex wird mit einem Wert zwischen 0 und 100 angegeben, wobei eine Zahl nahe bei 0 ein sehr schwieriges Item darstellt, dass nur von sehr wenigen richtig beantwortet wurde und eine Zahl nahe bei 100 ein sehr einfaches Item darstellt, welches von fast allen richtig gelöst wurde. Für die Berechnung des Schwierigkeitsindex der Benennaufgabe wurden die Antworten der Versuchspersonen kodiert. Für eine korrekte Benennung eines Items wurde der Wert 1 vergeben und für unkorrekte der Wert 0. Die Ergebnisse des semantischen Entscheidungstest waren bereits durch das verwendete Programm (E-Prime® 2.0; © 2007 Psychology Software Tools, Inc.) dichotom kodiert.

Aufgrund der Tatsache, dass Reaktionszeiten nicht dichotom kodiert werden können, ist eine Berechnung des Schwierigkeitsindex wie oben beschrieben für diesen Wert nicht möglich. Da jedoch die Reaktionszeit für eine Itembenennung oder für eine Entscheidung bereits selbst eine qualitative Aussage über die Schwierigkeit eines Items darstellt (je länger die Reaktionszeit, desto schwieriger das Item), konnte ein weiterer Wert berechnet werden, welcher sich aus dem Mittelwert der Reaktionszeiten aller Versuchspersonenscores über ein Item ergibt. Um Ausreisserantworten mit sehr hohen bzw. sehr niedrigen Reaktionszeiten weniger stark zu gewichten, wurde für die Berechnungen der Median anstelle des Mittelwerts verwendet. Die berechneten Schwierigkeitsindexen und die Mediane der Reaktionszeiten ergaben starke Korrelationen für den Benenntest, $r_s = -.720$, $p < .001$, und für den semantischen Entscheidungstest, $r_s = -.590$, $p < .001$. Demnach gingen schwierigere Bilder des Benenntests sowie schwierigere Bild-Wort-Paare mit längeren Reaktionszeiten einher.

Normalerweise sollte die Endversion eines Tests eine Mischung aus schwierigen, mittelschwierigen und einfachen Items enthalten, um zwischen Personen mit unterschiedlichen Merkmalsausprägungen gut differenzieren zu können (Fisseni, 1997). Da die aktuelle Untersuchung jedoch mit sprachgesunden Personen durchgeführt wurde und davon ausgegangen werden kann, dass Items, welche bereits für diese Personen schwierig sind, für Patienten ebenso schwierig beziehungsweise nicht mehr lösbar sind, wurde bei der Itemauswahl darauf geachtet, dass nur die Items in die Endversion gelangen, welche durch die Benennaufgabe einen Schwierigkeitsindex nahe 100, jedoch von mindestens 50 aufwiesen. Die Itemanalyse führte zur Entfernung von sechs Wort- bzw. Bildpaare (Marder – Biber, Walross – Seelöwe, Pfirsich – Zwetschge, Zigarre – Zigarette, Brosche – Spange und Ball – Dartpfeil) aus den definitiven Testitems, da die Bilder wegen der fehlenden Farbe oder der generellen Bildqualität nicht eindeutig von den Versuchspersonen erkannt werden konnten. Somit wurden 29 biologische und 29 manipulierbare Wort- bzw. Bildpaare für die Erstellung der finalen Testversionen verwendet. Dies bedeutet, dass die Parallelversionen des Benenntests jeweils 58 Items und die Parallelversionen des semantischen Entscheidungstests jeweils 116 Items umfassen.

Bennentest

Dominante Antwort

Um die definitive Bezeichnung für jedes Item zu erhalten, wurde jeweils die Antwort ausgewählt, die von den meisten Versuchspersonen gegeben wurde und somit die dominante Antwort darstellt. Hierfür wurden bewusst die Antworten der Benennaufgabe und nicht die nochmalige Benennung der Bilderbeurteilung verwendet, da die Bildbenennungen der Beurteilungsaufgabe durch die vorangegangene Aktivierung der einzelnen Konzepte in der Benenn- und Entscheidungsaufgabe beeinflusst sein könnten.

Zusammenhang & Unterschiede der demographischen Angaben der Vpn auf die Reaktionszeit und die Accuracy des Benenntests

Um festzustellen, ob das Alter, das Geschlecht und die Anzahl Bildungsjahre einen Einfluss auf die Reaktionszeit (Median) und die Accuracy im Benenntest haben, wurden Korrelationsberechnungen vorgenommen. Das Alter der Versuchspersonen korrelierte hoch signifikant mit der Reaktionszeit von biologischen Objekten, $r_s = .279$, $p < .001$, was bedeutet, je älter die Versuchsperson war, desto mehr Zeit wurde für die Benennung benötigt. Dieser Effekt wurde zudem durch einen signifikanten Mann-Whitney *U*-Test bestätigt, welcher ergab, dass Versuchspersonen unter 30 Jahren die Bilder generell signifikant schneller benannten, als Versuchspersonen ab 30 Jahren, $U = 223$, $Z = -2.105$, $p = .035$. Des Weiteren korrelierten die Bildungsjahre mit der Accuracy aller Items, $r_s = .317$, $p < .001$, und mit der Accuracy von biologischen Objekten, $r_s = .337$, $p < .001$. Dies bedeutet, je mehr Bildungsjahre eine Versuchsperson absolvierte, desto mehr Bilder wurden korrekt benannt. Alle Korrelationskoeffizienten sind in der Tabelle 2 und die Ergebnisse der Mann-Whitney *U*-Tests in der Tabelle 3 ersichtlich.

Summenscore als generelles Schwierigkeitsmass der Items

Um ein generelles Mass für die Schwierigkeit von jedem Item zu erhalten, wurde aus der Reaktionszeit (Median), dem Schwierigkeitsindex sowie allen erhobenen Parametern ein Summenscore gebildet. Das Item mit dem höchsten Summenscore stellt dabei das schwierigste, das Item mit dem tiefsten Summenscore das einfachste Testitem dar. Hierfür, und auch für die weiteren statistischen Auswertungen, wurden die Werte des Schwierigkeitsindex, der Bildübereinstimmung und der Vertrautheit umgepolt, sodass die Werte aller erfassten Parameter in die gleiche Richtung gehen (je höher der Wert, desto schwieriger). Anhand dieses Summenscores wurden später die Items in den Paralleltestversionen angeordnet.

Zusammenhang & Unterschiede der Parameter auf die Reaktionszeit des Benenntests

Zunächst sollte herausgefunden werden, welche der einbezogenen Parameter (Naming Agreement, Wortfrequenz, Wortlänge (Silben und Buchstaben), Wortkomplexität, Initialfrikation, Objektklasse, Bildübereinstimmung, Vertrautheit, Visuelle Komplexität) die Benennleistung der Probanden beeinflusst. Besteht eine signifikante Korrelation zwischen einem Parameter und der Reaktionszeit, ist anzunehmen, dass dieser Parameter einen bedeutsamen Effekt auf die Benennleistung hat. Besteht ein solcher Effekt bereits bei einer gesunden Population, so kann davon ausgegangen werden, dass dieser bei einer Patientenpopulation mindestens gleich gross oder grösser ist. Die Daten der Parameter wurden mit dem Kolmogorov-Smirnoff-Test auf ihre Verteilung untersucht. Da lediglich die Daten der Vertrautheit und der visuellen Komplexität normalverteilt waren, wurden ausschliesslich nichtparametrische Test für die Berechnungen verwendet. Die Korrelationskoeffizienten aller Parameter auf die Reaktionszeit sind in der Tabelle 4 dargestellt. Es ergaben sich signifikante Korrelationen zwischen der Reaktionszeit und der Wortfrequenz, $r_s = .372$, $p < .001$, dem Naming Agreement, $r_s = .362$, $p < .001$, der Wortkomplexität, $r_{pb} = .194$, $p < .05$, sowie der Bildübereinstimmung, $r_s = .560$, $p < .001$.

Ferner wurde analysiert, ob sich die Effekte der Parameter zwischen den biologischen und den manipulierbaren Objekten unterscheiden (siehe Tabelle 5). Mann-Whitney *U*-Tests ergaben signifikant unterschiedliche Effekte der beiden Objektklassen bei den Parametern Wortlänge (Buchstaben), $U = 1269.5$, $Z = -2.313$, $p = .021$, Naming Agreement, $U = 1122$, $Z = -3.288$, $p < .001$, Bildübereinstimmung, $U = 1079.5$, $Z = -3.329$, $p < .001$, und Vertrautheit, $U = 907.5$, $Z = -4.277$, $p < .001$. Manipulierbare Objekte bestanden demnach aus mehr Buchstaben und erzielten ein tieferes Naming Agreement als biologische Objekte, jedoch war die Bildübereinstimmung und die Vertrautheit von manipulierbaren Objekten höher als die der biologischen Objekten.

Erstellung der Paralleltestversionen des Benenntests

Die Wortpaare wurden auf zwei provisorische Paralleltestversionen verteilt, indem jeweils das erste Wort eines Paares in die Version 1 und das zweite Wort eines Paares in die Version 2 eingefügt wurde. Danach wurden die Testitems der beiden Versionen entlang des Summenscores aufsteigend angeordnet. Um sicherzustellen, dass die Items in den beiden Testversionen insbesondere auf den Summenscore bezogen, jedoch auch auf alle durch die Korrelation als bedeutsam erwiesenen Parameter, gleichmässig verteilt sind, wurde ein Wilcoxon-Test durchgeführt. Jegliche Unterschiede zwischen den beiden Testversionen wurden durch Umtauschen der Wörter eines Wortpaares und erneuter Kontrolle mit dem Wilcoxon-Test bereinigt, bis sich die beiden Testversionen bezüglich der Summenscores und der bedeutsamen Korrelationen nicht mehr unterschieden. Die Ergebnisse des Wilcoxon-Tests für die finalen Paralleltestversionen sowie des McNemar χ^2 Tests für die dichotomen Parameter Objektklasse und Wortkomplexität sind in der Tabelle 6 dargestellt. Die Reliabilitätsmessung, welche in der Tabelle 7 ersichtlich ist, ergab ausgezeichnete interne Konsistenzen für beide Paralleltestversionen (Crombach's α = .998). Die finalen Paralleltestversionen sind im Anhang A und B und die dazugehörigen Daten der Parameter in den Tabellen 8 und 9 dargestellt.

Semantischer Entscheidungstest

Zusammenhang & Unterschiede der demographischen Angaben der Vpn auf die Reaktionszeit und die Accuracy des semantischen Entscheidungstests

Die Korrelationsergebnisse zwischen der Reaktionszeit (Median) bzw. Accuracy und dem Alter, Geschlecht, Anzahl Bildungsjahre und Händigkeit der Versuchspersonen sind in der Tabelle 10 dargestellt. Das Alter der Versuchspersonen korrelierte signifikant mit der Reaktionszeit, r_s = .633, p < .001. Dies bedeutet, je älter eine Versuchsperson war, desto

mehr Zeit wurde für die Entscheidung benötigt. Dieser Effekt konnte ebenfalls mit einem Mann-Whitney *U*-Test bestätigt werden (siehe Tabelle 11), welcher ergab, dass Versuchspersonen unter 30 Jahren die Entscheidungen signifikant schneller trafen, als Versuchspersonen ab 30 Jahren, $U = 111$, $Z = -4.154$, $p < .001$. Zudem bestanden für die Reaktionszeit signifikante Korrelationen zwischen manipulierbaren Objekten, $r_{pb} = .284$, $p < .05$, sowie konsistenten Bild-Wort-Paaren, $r_{pb} = .283$, $p < .05$, mit der Händigkeit. Mann-Whitney *U*-Tests ergaben (siehe Tabelle 12), dass Rechtshänder manipulierbare Objekte signifikant, $U = 22$, $Z = -2.021$, $p = .043$, und konsistente Bild-Wort-Paare tendenzielle schneller, $U = 25$, $Z = -1.903$, $p = .057$, entschieden als Linkshänder.

Zusammenhang & Unterschiede der Parameter Kongruenz und Objektklasse auf die Reaktionszeit und die Accuracy des semantischen Entscheidungstests

Ein erster Vergleich der Reaktionszeit und der Accuracy der Items im semantischen Entscheidungstest unter Berücksichtigung des Parameters Kongruenz zeigt, dass die Reaktionszeiten von kongruenten Bild-Wort-Paaren im Durchschnitt kürzer waren (Mdn = 1040) und häufiger eine korrekte Entscheidung getroffen wurden (Accuracy $M = 50.74$, SD = 2.073), als diejenigen von inkongruenten Bild-Wort-Paaren (Reaktionszeit $Mdn = 1155$; Accuracy $M = 48.40$, $SD = 4.185$). Des Weiteren ist unter Berücksichtigung des Parameters Objektklasse ersichtlich, dass Bild-Wort-Paare von manipulierbaren Objekten im Durchschnitt mit kürzeren Reaktionszeiten ($Mdn = 1097$) und weniger korrekten Entscheidungen einhergingen (Accuracy $M = 49.32$, $SD = 3.846$), als Bild-Wort-Paare von biologischen Objekten (Reaktionszeit $Mdn = 1103$, Accuracy $M = 49.82$, $SD = 3.108$). Um herauszufinden, ob diese Unterschiede der Kongruenz und Objektklasse signifikant sind, wurde jeweils für die Reaktionszeit und die Accuracy eine zweifaktorielle Varianzanalyse Kongruenz (kongruent, inkongruent) x Objektklasse (biologisch, manipulierbar) mit Messwiederholung auf beiden Faktoren gerechnet. Die Ergebnisse der zweifaktoriellen

Varianzanalyse sind für die Reaktionszeit in der Tabelle 13 und für die Accuracy in der Tabelle 14 ersichtlich. Beim Faktor Kongruenz trat ein Haupteffekt für die Reaktionszeit, F (1, 57) = 62.701, $p < .001$, und die Accuracy, F (1, 57) = 29.456, $p < .001$, auf. Der Faktor Objektklasse hingegen wurde für die Reaktionszeit und die Accuracy nicht signifikant. Es ergab sich eine signifikante Interaktion der beiden Faktoren für die Reaktionszeit, F (1, 57) = 14.525, $p < .001$, jedoch nicht für die Accuracy, F (1, 57) = 1.271, $p = .264$.

Erstellung der Paralleltestversionen des semantischen Entscheidungstests

Für die Erstellung der Parallelversionen des semantischen Entscheidungstest wurde die genau gleiche Aufteilung der Testitems auf die beiden Parallelversionen vorgenommen, wie sie durch den Wilcoxon-Test beim Benenntest zustande gekommen ist. Um jedoch zu vermeiden, dass zuerst alle Testitems von den leichteren kongruenten Bild-Wort-Paaren manipulierbaren Objektklasse und erst danach alle Testitems von den schwierigeren inkongruenten Bild-Wort-Paaren der biologischen Objektklasse aufgeführt werden, wurden die Testitems nicht aufsteigend entlang ihrer generellen Schwierigkeit, sondern randomisiert angeordnet, sodass sich eine gute Durchmischung der Testitems ergab. Die finalen Paralleltestversionen sind im Anhang C und D und die dazugehörigen Daten der Parameter in den Tabellen 15 und 16 ersichtlich.

Diskussion

Ziel der vorliegenden Arbeit war die Normierung und die Erstellung von Benenn- und semantischen Entscheidungstests für biologische und manipulierbare Objektbilder, welche für die Parameter Wortlänge (Silben und Buchstaben), Wortfrequenz, Naming Agreement, Wortkomplexität, Initialfrikation, Bildübereinstimmung, Vertrautheit, visuelle Komplexität, Objektklasse sowie Kongruenz kontrolliert sind. Die Datenanalyse des Benenntests hat ergeben, dass die Wortfrequenz, das Naming Agreement, die Wortkomplexität und die

Bildübereinstimmung einen signifikanten Einfluss auf die Reaktionszeit haben. Bei der statistischen Analyse der Entscheidungsaufgabe ergab sich ein signifikanter Haupteffekt des Faktors Kongruenz für die Reaktionszeit und die Accuracy. Des Weiteren wurde für die Reaktionszeit eine signifikante Interaktion der beiden Faktoren Kongruenz und Objektklasse ersichtlich. Anhand dieser Befunde wurden zwei Paralleltestversionen eines Benenntest mit gleicher Anzahl von biologischen und manipulierbare Objekten erstellt, welche für die als bedeutsam erwiesenen Parameter und einen zusätzlich erstellten Summenscore kontrolliert sind. Die Erstellung der beiden Paralleltestversionen des semantischen Entscheidungstests erfolgte mit der gleichen Itemaufteilung wie beim Benenntest, wobei die Items jeweils randomisiert angeordnet wurden.

Die Analyse der demographischen Angaben der Versuchspersonen und der Reaktionszeiten bzw. der Accuracy ergab, dass Versuchspersonen unter 30 Jahren die Bilder signifikant schneller benannten sowie signifikant schneller Entscheidungen getroffen haben, als Versuchspersonen ab 30 Jahren. Dieser Unterschied wird durch den normalen kognitiven Alterungsprozess erklärt, welcher die kognitive Verarbeitungsgeschwindigkeit mindern kann (vgl. Mayr, 2006). Der positive Zusammenhang zwischen den Bildungsjahren und der Accuracy könnte dadurch erklärt werden, dass mit steigender Anzahl an Bildungsjahren auch eine bessere Differenzierung von ähnlichen Konzepten ermöglicht wird. Des Weiteren zeigte sich bei der semantischen Entscheidungsaufgabe, dass Rechtshänder die Entscheidungen schneller getroffen haben, als Linkshänder. Da allerdings die Vergleichbarkeit der beiden Gruppen (48 Rechtshänder und 3 Linkshänder) infrage gestellt werden muss, müsste dieses Ergebnis mit ausgeglichenen Gruppen nochmals überprüft werden.

Linguistische Parameter

Der Einfluss, welcher die Wortfrequenz auf die Reaktionszeit hat (bspw. Oldfield & Wingfield, 1965; Jescheniak & Levelt, 1994; Barry, Morrison & Ellis, 1997; Bates et al., 2003), konnte auch in dieser Studie repliziert werden. Je tiefer die Frequenzklasse eines Wortes ist, desto häufiger wird es in der Alltagssprache verwendet und kann demnach auch schneller abgerufen werden. Szekely et al. (2003) konnten diesen Einfluss nur bei Objektbildern, jedoch nicht bei Tätigkeitsbildern feststellen und führten dies als eine mögliche Erklärung auf die statische Darstellung der Tätigkeitsbilder zurück. Die in der aktuellen Studie verwendeten manipulierbaren und biologischen Objekte zeigten keine Unterschiede des Einflusses der Frequenz auf die Reaktionszeit. Möglicherweise kann durch die Verwendung von manipulierbaren Objekten anstelle von Tätigkeitsbildern die bisherige Problematik der Darstellung von Tätigkeiten umgangen werden.

In Übereinstimmung mit früheren Studien (bspw. Bates et al., 2003; Barry et al., 1997; Genzel et al., 1995) wurde für das Naming Agreement ein signifikant positiver Zusammenhang mit der Reaktionszeit gefunden. Im Gegensatz zu einem tiefen Naming Agreement, führte ein hohes Naming Agreement zu kürzeren Reaktionszeiten für die Benennung eines Objekts. Diese Befunde können dadurch erklärt werden, dass bei einem Objekt mit tiefem Naming Agreement von unterschiedlichen aktivierten Bezeichnungen die passendste ausgewählt werden muss und dafür mehr Zeit benötigt wird. Objekte mit hohem Naming Agreement aktivieren hingegen nur wenige, bzw. nur eine Bezeichnung, wodurch der Auswahlprozess deutlich weniger aufwendig ist und somit mit einer kürzeren Reaktionszeit einhergeht.

Des Weiteren zeigte die getrennte Analyse des Naming Agreements für biologische und manipulierbare Objekte einen signifikanten Unterschied, bei welchem biologische Objekte durchschnittlich ein höheres Naming Agreement als manipulierbare Objekte

aufwiesen. Eine mögliche Erklärung für den Naming Agreement Unterschied ist, dass für biologische Objekte häufig nur wenige Bezeichnungen zu Verfügung stehen, um es eindeutig zu benennen und daher ein höheres Naming Agreement zustande kommt. Hingegen können manipulierbare Objekten häufig durch verschiedene Synonyme eindeutig benannt werden, was zu einem tieferen Naming Agreement führt (bspw. Zapfenzieher, Synonym: Korkenzieher; Füllfeder, Synonym: Füller, Feder, Füllfederhalter).

Wie bereits in früheren Studien belegt wurde (siehe D'Amico et al., 2001; Szekely et al., 2005; Bates et al, 2003), zeigte sich auch in dieser Studie ein signifikanter Zusammenhang zwischen der Wortkomplexität und der Reaktionszeit. Im Vergleich mit dem Einfluss des Naming Agreements und der Wortfrequenz war dieser Einfluss sehr klein und stellt daher nur eine sehr schwache Korrelation dar. Zudem wurde in dieser Studie der positive Zusammenhang zwischen der Wortkomplexität mit der Wortfrequenz und der Wortlänge (Buchstaben und Silben) repliziert, indem komplexe Wörter länger und mehrsilbiger sind, sowie weniger häufig vorkommen, als einfache Wörter. Für die Wortlänge (Silben und Buchstaben) konnte kein Zusammenhang mit der Latenzzeit für das Benennen festgestellt werden. Die Resultate der vorliegenden Arbeit divergieren mit Ergebnissen anderer Studien (bspw. Szekely et al., 2005, D'Amico et al., 2003), welche mindestens kleine Effekte feststellen konnten. Ungeachtet dessen ergab sich ebenfalls der Zusammenhang zwischen der Wortfrequenz und der Wortlänge (Silben und Buchstaben), welcher dem Zipfschen Gesetz entspricht. Somit waren auch in dieser Studie hochfrequente Wörter generell kürzer als tieffrequente Wörter, welche häufig mehrsilbig waren und aus vielen Buchstaben bestanden.

Bei früheren Studien (bspw. Szekely et al., 2005, Bates et al. 1995, Bates et al., 2003) wurden Zusammenhänge zwischen Wörtern, welche mit einer Initalfrikation beginnen, und der Latenzzeit zur Benennung festgestellt. Dabei wurden Antworten aus den Auswertungen

ausgeschlossen, welche aufgrund dieser Reibelaute zu einer künstlichen Verlängerung der Reaktionszeiten führten. In der vorliegenden Studie wurde eine manuelle Anpassung dieser künstlich verlängerten Reaktionszeiten vorgenommen, damit diese Wörter mit den restlichen Wörtern vergleichbar blieben und nicht aus den Auswertungen ausgeschlossen werden mussten. Da sich kein Zusammenhang zwischen der Initialfrikation und der Reaktionszeit ergab, konnte diese Problematik durch die differente Herangehensweise gelöst werden.

Bildparameter

Gemäss früheren Studien führen eine hohe Bildübereinstimmung, eine hohe Vertrautheit mit dem Konzept und eine tiefe visuelle Komplexität des Bildes zu kürzeren Reaktionszeiten (Snodgrass & Vanderwart, 1980; Genzel et al., 1995; Ellis & Morrison, 1998). Von den drei erfassten Bildparametern erwies sich lediglich die Bildübereinstimmung als signifikanter Einfluss auf die Reaktionszeit. Die Vertrautheit mit einem Konzept und die visuelle Komplexität eines Bildes standen im Gegensatz zu den früheren Studien nicht im Zusammenhang mit der Reaktionszeit. Des Weiteren ergaben sich, in Übereinstimmung mit früheren Studien, Interkorrelationen der Bildparameter. Die visuelle Komplexität korrelierte signifikant mit der Vertrautheit. Demnach waren visuell weniger komplexe Bilder stärker vertraut, als visuell komplexere Bilder. Die Bildübereinstimmung korrelierte ebenfalls signifikant mit der Vertrautheit eines Konzepts, was bedeutet, dass eine höhere Bildübereinstimmung mit einer stärkeren Vertrautheit einhergeht und somit vertrautere Objekte häufig übereinstimmende prototypische Bilder aktivieren.

Hierfür ergaben sich ebenfalls signifikante Unterschiede zwischen manipulierbaren und biologischen Objekten. Die Bildübereinstimmung und die Vertrautheit waren für manipulierbare Objekte signifikant höher als für biologische Objekte. Dieser Unterschied in der Bildübereinstimmung kann durch die schwarz-weissen Strichzeichnungen erklärt werden.

Während die Farbe die Prototypikalität von manipulierbaren Objekten mindern kann, da unterschiedliche Personen unterschiedliche Farben damit assoziieren, gibt es bei vielen biologischen Objekten eine einzige damit assoziierte Farbe (vgl. Price & Humphreys, 1989; Rossion & Pourtois, 2004). Sind die Bilder nicht farbig dargestellt, wird somit die Bildübereinstimmung der biologischen Objekte gemindert. Eine mögliche Erklärung für die unterschiedliche Vertrautheit der beiden Objekte ist, dass die Vertrautheit von manipulierbaren Objekten aufgrund deren alltäglichen Konfrontation (bspw. Messer und Gabel) höher eingeschätzt wird, als biologische Objekte, welche zwar vertraut sind, jedoch nicht täglich konfrontiert werden (bspw. Tiere auf einem Bauernhof).

Kategorienspezifische Parameter

Aufgrund der sprachgesunden Normierungsstichprobe wurde nicht erwartet, dass sich Schwierigkeiten in der Benennung von biologischen oder manipulierbaren Objekten ergeben würden. In Übereinstimmung mit dieser Hypothese wurden weder Schwierigkeiten in der Benennung, noch signifikante Unterschiede der Latenzzeit für die Benennung gefunden. Ebenso ergaben sich bei der semantischen Entscheidungsaufgabe keine Unterschiede oder Schwierigkeiten für die Reaktionszeit und die Korrektheit der Entscheidungen für biologische und manipulierbare Objekte. Wie jedoch bereits erwähnt, kann der Abruf von belebten bzw. unbelebten Objekten selektiv beeinträchtigt sein. Da die beiden Tests für den klinischen Gebrauch über verschiedene Patientengruppen hinweg erstellt werden sollten, ist es unabdingbar solche Beeinträchtigungen feststellen zu können, da sie essenziell für den weiteren Therapieverlauf sind. Aus diesem Grund wurde der dichotome Parameter Objektklasse für die Konstruktion der Tests weiter mitberücksichtigt.

Gemäss Gellatly und Gregg (1975) wird für eine korrekte Entscheidung bei inkongruenten semantisch ähnlichen Bild-Wort-Paaren mehr Zeit benötigt, als bei

kongruenten semantisch ähnlichen Bild-Wort-Paaren. Diese unterschiedliche Verarbeitungszeit konnte in der vorliegenden Studie erneut durch den signifikanten Haupteffekt Kongruenz bei der semantischen Entscheidungsaufgabe belegt werden. Zudem waren die Entscheidungen bei kongruenten Paaren häufiger korrekt als bei inkongruenten Paaren. Aufgrund der semantischen, phonologischen oder funktionalen Ähnlichkeit oder der Assoziationen der Stimuli-Paare benötigte es für inkongruente Paare mehr Zeit für eine korrekte Entscheidung, weil eine Vielzahl an Vergleichen getätigt werden muss, damit die Unterschiede erkannt werden. Die höhere Fehleranzahl bei inkongruenten Paaren kann durch einen unvollständigen Vergleichsprozess erklärt werden, bei welchem man sich durch die hohe Ähnlichkeit dazu verleiten lässt, eine Kongruenz anzunehmen. Beispielsweise wird das Bild „Erdnuss" mit dem Wort „Erdbeere" präsentiert. Wird nur der Anfang des geschriebenen Wortes wahrgenommen, kann dies zu einer Kongruenz-Entscheidung führen, da eine Übereinstimmung der beiden Stimuli angenommen wird. Oder bei einer hohen visuellen Ähnlichkeit (beispielsweise Wolf und Fuchs) kann es zu fehlerhaften Entscheidungen führen, wenn keine vollständiger Merkmalsabgleich vorgenommen wird.

Des Weiteren ergab sich für die Reaktionszeit eine signifikante Interaktion der beiden Faktoren Kongruenz und Objektklasse. Während für kongruente manipulierbare Paare schneller entschieden werden konnte als für kongruente biologische Paare, benötigte die Entscheidung bei inkongruenten manipulierbaren Paaren mehr Verarbeitungszeit als bei inkongruenten biologischen Paaren. Die Identifizierung von manipulierbaren Objekten über ihre Funktion führte demnach bei kongruenten Paaren zu kürzeren Reaktionszeiten als die Identifizierung von biologischen Objekten über die sensorischen Merkmale (vgl. hierzu auch Woollams et al. 2008; Martin & Chao, 2001). Jedoch scheint die Unterscheidung von funktional ähnlichen manipulierbaren Paaren mit einem noch grösseren Verarbeitungsaufwand in Verbindung zu stehen als für die Identifizierung von biologischen

Objekten, was zu den längeren Reaktionszeiten der inkongruenten manipulierbaren Paaren im Vergleich zu den biologischen Paaren führte.

Aufgrund der Tatsache, dass diese Entscheidungsaufgaben einen hohen Verarbeitungsaufwand benötigen, bei welchen eine Vielzahl von Informationen abgerufen und verarbeitet werden müssen und wie durch die Studie gezeigt, für sprachgesunden Personen eine schwierige Aufgabe mit Unterschieden in der Reaktionszeit und der Accuracy darstellt, ist davon auszugehen, dass für sprachbeeinträchtigte Patienten solche Aufgaben mindestens ebenso schwierig bis, je nach Ätiologie, nicht mehr lösbar sind und somit bedeutende Hinweise für die Diagnosestellung und den weiteren Therapieverlauf geben können.

Limitationen und Fazit

Eine Limitation der vorliegenden Studie betrifft die Durchführung der Untersuchung in hochdeutscher Sprache. Die Untersuchung wurde ausschliesslich mit Personen durchgeführt, welche in ihrem Alltag in einem schweizerdeutschen Dialekt kommunizieren. Da sich die Phonologie gewisser schweizerdeutschen und hochdeutschen Wörter mit der gleichen Bedeutung teilweise sehr unterscheiden können (bspw. die hochdeutschen Wörter Kartoffel und Karotte: schweizerdeutsch: Härdöpfel und Riebli) ist es möglich, dass der Abruf des hochdeutschen Wortes teilweise mit einer längeren Reaktionszeit verbunden ist, als bei einem Abruf im Dialekt. Dadurch würde die Schwierigkeit der Items überschätzt, was unter Umständen zur Konstruktion von zu einfachen Tests führen könnte. Eine Möglichkeit dies zu überprüfen, wäre die erneute Durchführung an einer Hochdeutsch sprechenden Stichprobe um allfällige Unterschiede der Reaktionszeiten feststellen zu können.

Zudem erwiesen sich nahezu alle Items für die Normierungsstichprobe als sehr einfache Items. Da von der Annahme ausgegangen wurde, dass Items, welche für

Sprachgesunde mittelschwierig und schwierig sind, für Patienten mindestens genauso schwierig bis unlösbar sind, sollten für die Testkonstruktion nur einfache Items verwendet werden. Die dadurch sehr wenigen auszuschliessenden Items führten zu Paralleltests, welche für ein Diagnoseverfahren im klinischen Alltag zu viele Items umfassen. Erst durch die Untersuchung mit Patienten mit Wortfindungsstörungen aufgrund unterschiedlicher Ätiologie wird eine Unterteilung in einfache, mittelschwierige und schwierige Items möglich sein und damit auch eine akkurate Auswahl der Testitems, sodass die einzelnen Paralleltests eine angemessene Itemanzahl umfassen.

Zusammenfassend konnte in dieser Studie ein Benenn- und ein semantischer Entscheidungstest für biologische und manipulierbare Objekte erstellt werden, welche für die Parameter Wortfrequenz, Naming Agreement, Wortkomplexität, Bildübereinstimmung, Objektklasse sowie Kongruenz kontrolliert worden sind. Aufgrund der Normierung in deutscher Sprache können Erkenntnisse über das Benennen von biologischen und manipulierbaren Objekte gesammelt, sowie auch durch den semantischen Entscheidungstest die Differenzierungsfähigkeit zwischen ähnlichen Konzepten (semantisch bzw. phonologisch Ähnlichkeit und Assoziationen bei biologischen Objekten und ähnliche Funktionen bei manipulierbaren Objekten) erfasst werden. Es besteht die Hoffnung, dass diese Tests Wortfindungsstörungen über verschiedene Patientengruppen und unterschiedliche Beeinträchtigungsgrade sensibel erfassen können und somit eine Bereicherung für die im klinischen Alltag verwendeten Diagnoseverfahren darstellen.

Literaturverzeichnis

Badre, D., & Wagner, A.D. (2007). Left ventrolateral prefrontal cortex and the cognitive control of memory. *Neuopsychologia, 45*, 2883-2901.

Barry, C., Morrison, C.M., & Ellis, A.W. (1997). Naming the Snodgrass and Vanderwart Pictures: Effects of Age of Acquisition, Frequency, and Name Agreement. *The Quarterly Journal of Experimental Psychology, 50A* (3), 560-585.

Bates, E., D'Amico, S., Jacobsen, T., Szekely, A., Andonova, E., Devescovi, A., et al. (2003). Timed picture naming in seven languages. *Psychonomic Bulletin & Review, 10* (2), 344-380.

Bates, E., Marchman, V., Thal, D., Fenson, L., Dale, P., Reznick, J.S., et al. (1994). Developmental and stylistic variation in the composition of early vocabulary. *Journal of Child Language*, 21, 85-124.

Bates, E., Devescovi, A., Pizzamiglio, L., D'Amico, S., & Hernandez, A. (1995). Gender and lexical access in Italian. *Perception & Psychophysics, 57*, 847-862.

Baumann, T., Tolnay, M., & Monsch, A. (2009). Primär progressive Aphasie: Erinnern ohne Sprache. *Schweizerisches Medizin-Forum, 9* (37), 646-650.
Retrieved from the web 12.4.2012: http://www.medicalforum.ch

Bird, H., Howard, D., & Franklin, S. (2000). Why is a verb like an inanimate object? Grammatical category and semantic category deficits. *Brain and Language, 72*, 246-309.

Blanken, G., Döppler, R., & Schlenk, K.J. (1999). *Wortproduktionsprüfung.* Hofheim: NAT-Verlag.

Broca, P. (1861). Remarque sur le siège de la faculté de langage articulé, suivies d'une observation d'aphémie (perte de la parole). *Bulletins de la Sociéte d'anatomie (Paris), 6*, 330-357.

Brown, R., & McNeill, D. (1966). The „tip-of-the-tongue-phenomenom“. *Journal of Verbal Learning and Verbal Behavior, 5,* 325-337.

Chao, L.L., Haxby, J.V., & Martin, A. (1999). Attribute-based neural substrates in temporal cortex for perceiving and knowing about objects. *Nature Neuroscience, 2,* 913-919.

Chao, L.L., & Martin, A. (2000). Rapid communication. Representation of manipulable man-made objects in the dorsal stream. *NeuroImage, 12,* 478-484.

Croot, K. (2009). Progressive language impairments: Definitions, diagnoses, and prognoses. *Aphasiology, 23* (2), 302-326.

D’Amico, S., Devescovi, A., & Bates, E. (2001). Picture naming and lexical access in Italian children and adults. *Journal of Cognition & Development, 2,* 71-105.

De Bleser, R. (2006). Aufbau und Funktionen der Sprache. In Karnath, H.O. & Thier, P. (Hrsg.), *Neuropsychologie* (2. Auflage, S.340-345). Heidelberg: Springer Medizin Verlag.

De Bleser, R., Cholewa, J., Stadie, N., & Tabatabaie, S. (2004). *LeMo – Lexikon modellorientiert. Einzelfalldiagnostik bei Aphasie, Dyslexie und Dysgraphie.* München: Urban & Fischer.

Delavier, C. & Graham, A. (1981). *Basel-Minnesota Test zur Differentialdiagnose der Aphasie.* Basel: Institut für Sprache und Stimmtherapie, Kantonsspital Basel.

DeLeon, J., Gottesman, R., Kleinman, J.T., Newhart, M., Davis, C., Heidler-Gary, J., et al. (2007). Neural regions essential for distinct cognitive processes underlying picture naming. *Brain, 130,* 1408-1422.

Ellis, A. W., & Morrison, C. M. (1998). Real age-of acquisition effects in lexical retrieval. *Journal of Experimental Psychology: Learning, Memory, and Cognition, 24,* 515-523.

Fisseni, H.-J. (1997). *Lehrbuch der psychologischen Diagnostik. Mit Hinweisen zur Intervention.* 2. Auflage. Göttingen: Hogrefe.

Gellatly, A.R.H., & Gregg, V.H. (1975). The effects of negative relatedness upon word-picture and word-word comparisons and subsequent recall. *British Journal of Psychology, 66* (3), 311-323

Genzel, S., Kerkhoff, G., & Scheffter, S. (1995). PC-gestützte Standardisierung des Bildmaterials von Snodgrass & Vanderwart (1980). *Neurolinguistik, 9* (1), 41-53.

Goodglass, H. & Kaplan, E. (1972). *The Assessment of Aphasia and Related Disorders.* Philadelphia: Lea & Febiger.

Hodges, J.R., Patterson, K., Graham, N., & Dawson, K. (1996). Naming and knowing in dementia of Alzheimer's type. *Brain and Language, 54,* 302-325.

Hoffmann, A. (2010). *Normierung eines linguistisch basierten Benenntests für Objekte und Tätigkeiten in Deutscher und Schweizerdeutscher Sprache nach den Parametern Reaktionszeit bei Gesunden, Wortlänge, Wortfrequenz, Naming Agreement, Wortkomplexität, Initialfrikation und Belebtheit.* Unveröffentlichte Masterarbeit, Universität Bern.

Huber, W., Poeck, K., & Weniger, D. (2002). Aphasie. In Hartje, W. & Poeck, K. (Hrsg.), *Klinische Neuropsychologie* (5. Auflage, S.93-173). Stuttgart: Thieme.

Huber, W., Poeck, K., Weniger, D., & Willmes, K. (1983). *Der Aachener Aphasie-Test.* Göttingen: Hogrefe.

Huber, W. & Ziegler, W. (2009). Störungen der Sprache und Sprechen. In Sturm, W., Herrmann, M. & Münte, T.F. (Hrsg.), *Lehrbuch der klinischen Neuropsychologie* (2. Auflage, S. 558-608). Heidelberg: Spektrum Akademischer Verlag.

Jescheniak, J.D. & Levelt, W.J.M. (1994). Word frequency effects in speech production: Retrieval of syntactic information and of phonological form. *Journal of Experimental Psychology: Learning, Memory, and Cognition, 20,* 824-843.

Kaplan, E., Goodglass, H., & Weintraub, S. (1983). *Boston Naming Test.* Philadelphia: Lea & Febiger.

Kay, J., Lesser, R., & Coltheart, M. (1992). *Psycholinguistic Assessment of Language Processing in Aphasia (PALPA).* Hove, UK: Lawrence Erlbaum.

Kertesz, A. (1982). *Western Aphasia Battery.* New York: Grune and Stratton.

Klapp, S.T., Anderson, W.G., & Berrian, R.W. (1973). Implicit speech in reading, reconsidered. *Journal of Experimental Psychology, 100*, 368-374.

Le Rhun, E., Richard, F., & Pasquier, F. (2005). Natural History of primary progressive aphasia. *Neurology, 65*, 887-891.

Levelt, J.M. (1989). *Speaking: From Intention to Articulation.* Cambridge, MA: The MIT Press.

Levelt, J.M., Roelofs, A., & Meyer, A.S. (1999). A theory of lexical access in speech production. *Behavioral and Brain Sciences, 22*, 1-38.

Martin, A., & Chao, L.L. (2001). Semantic memory and the brain: structure and processes. *Current opinion in Neurobiology, 11*, 194-201.

Mätzig, S., Druks, J., Masterson, J., & Vigliocco, G. (2009). Noun and verb differences in picture naming: Past studies and new evidence. *Cortex, 45*, 738-758.

Mayr, U. (2006). Normales kognitives Altern. In Karnath, H.O. & Thier, P. (Hrsg.), *Neuropsychologie* (2. Auflage, S. 678-686). Heidelberg: Springer Medizin Verlag.

Mesulam, M.-M. & Weintraub, S. (1992). Spectrum of primary progressive aphasia. *Bailliere's Clinical Neurology: International Practice and Research, 1* (3), 583-609.

Münte, T.F. (2009). Neuropsychologische Defizite bei Demenzerkrankungen. In Sturm, W., Herrmann, M. & Münte, T.F. (Hrsg.), *Lehrbuch der klinischen Neuropsychologie* (2. Auflage, S. 726-739). Heidelberg: Spektrum Akademischer Verlag.

Oldfield, R.C. & Wingfield, A. (1965). Response latencies in naming objects. *Quarterly Journal of Experimental Psychology, 17,* 273-281.

Poeck, K. & Hartje, W. (2002). Demenz. In Hartje, W. & Poeck, K. (Hrsg.), *Klinische Neuropsychologie* (5. Auflage, S.423-434). Stuttgart: Thieme.

Price, C.J. (2010). The anatomy of language: a review of 100 fMRI studies published in 2009. *Annals of the New York Academy of Sciences. Issue: The Year in Cognitive Neuroscience, 1191*, 62-88.

Price, C.J., & Humphreys, G.W. (1989). The effects of surface detail on object categorization and naming. *Quarterly Journal of Experimental Psychology, 41,* 797-828.

Reischies, F.M. (2006). Demenz. In Karnath, H.O. & Thier, P. (Hrsg.), *Neuropsychologie* (2. Auflage, S. 678-686). Heidelberg: Springer Medizin Verlag.

Rossion, B. & Pourtoise, G. (2004). Revisiting Snodgrass and Vanderwart’s object databank: color and texture improve object recognition. *Perception, 33*, 217-236.

Saffran, E.M., Schwartz, M.F., & Marin O.S.M. (1980). The word order problem in agrammatism: II. Production. *Brain and Language, 10*, 263-280.

Schuell, H. (1965). *Differential Diagnosis of Aphasia with the Minnesota Test.* Minneapolis: University of Minnesota Press.

Snodgrass, J. G. & Vanderwart, M. A. (1980). Standardized set of 260 pictures: Norms for naming agreement, familiarity and visual complexity. *Journal of Experimental Psychology, Human Learning and Memory, 6*, 174-215.

Szekely, A., D’Amico, S., Devescovi, A., Federmeider, K., Herron, D., Iyer, G., et al. (2005). Timed Action and Object Naming. *Cortex, 41,* 7-25.

Vigliocco, G., Antonini, T., & Garrett, M.F. (1997). Grammatical gender is on the Tip of Italian tongues. *Psychological Science, 8,* 314-317.

Vigneau, M., Beaucousin, V., Hervé, P.Y., Duffau, H., Crivello, F., Houdé, O., et al. (2006). Meta-analyzing left hemisphere language areas: Phonology, semantics, and sentence processing. *NeuroImage, 30*, 1414-1432.

Vinson, D.P., & Vigliocco, G. (2002). A semantic analysis of grammatical class impairments: semantic representations of object nouns, action nouns and action verbs. *Journal of Neurolinguistics, 15*, 317-351.

Weniger, D. (2006). Aphasien. In Karnath, H.O. & Thier, P. (Hrsg.), *Neuropsychologie* (2. Auflage, S. 356-372). Heidelberg: Springer Medizin Verlag.

Wernicke, C. (1874). *Der aphasische Symptomkomplex: Eine psychologische Studie auf anatomischer Basis.* Breslau: Cohn & Weigert.

Woollams, A. M., Cooper-Pye, E., Hodges, J. R., & Patterson, K. (2008). Anomia: A doubly typical signature of semantic dementia. *Neuropsychologia, 46,* 2503-2514.

Zingeser, L., & Berndt, R. (1990). Retrieval of verbs and nouns in agrammatism and anomia. *Brain and Language, 39,* 14-32.

Zipf, G.K. (1965). *Human Behavior and the Principle of Least Effort: an Introduction to Human Ecology.* New York: Hafner.

Tabelle 1

Verwendete Wortpaare im Benenn- und im semantischen Entscheidungstest

Nr.	Biologische Objekte		Manipulierbare Objekte (inkl. deren Manipulation/Verb)		
1	Apfel	Birne	Messer	Schere	schneiden
2	Eule	Ente	Lampe	Kerze	anzünden
3	Huhn	Gans	Kamm	Bürste	frisieren
4	Kürbis	Peperoni	Sieb	Trichter	filtrieren
5	Melone	Zitrone	Kreide	Füllfeder	schreiben
6	Pelikan	Pinguin	Mütze	Hut	auf den Kopf setzen
7	Schnecke	Schlange	Besen	Schaufel	kehren
8	Schwan	Storch	Gabel	Löffel	essen
9	Spargel	Lauch	Schnur	Seil	festmachen
10	Tiger	Löwe	Zigarette	Zigarre	rauchen
11	Zebra	Esel	Flöte	Trompete	blasen
12	Pfau	Hahn	Haken	Schraube	reinschrauben
13	Katze	Hase	Pinzette	Zange	rausziehen
14	Hirsch	Reh	Radiergummi	Schwamm	wegputzen
15	Raupe	Wurm	Bostitch	Locher	draufdrücken
16	Gurke	Erbse	Hobel	Raffel	abtragen
17	Wolf	Fuchs	Brosche	(Haar-)spange	festmachen
18	Zwiebel	Tomate	Schlüssel	Schloss	zumachen
19	Erdbeere	Erdnuss	Harfe	Gitarre	Saiten zupfen
20	Elefant	Krokodil	Glas	Tasse	trinken
21	Palme	Kaktus	Dosenöffner	Korkenzieher	öffnen
22	Pfirsich	Pflaume	Gewehr	Pistole	schiessen
23	Banane	Orange	Koffer	Rucksack	packen
24	Karotte	Kartoffel	Bleistift	Pinsel	aufzeichnen
25	Specht	Spinne	Wäscheklammer	Büroklammer	festmachen
26	Nashorn	Nilpferd	Staubsauger	Bügeleisen	hin- und herfahren
27	Schaf	Ziege	Schlittschuhe	Rollschuhe	fahren
28	Marder	Biber	Giesskanne	Gartenschlauch	bewässern
29	Pudel	Dackel	Trommel	Klavier	beidhändig Töne erzeugen
30	Schmetterling	Libelle	Gürtel	Kette	beidhändig schliessen
31	Seelöwe	Walross	Beil	Hammer	draufschlagen
32	Frosch	Känguru	Ball	Dartpfeil	werfen

Tabelle 2

Korrelationen zwischen den demographischen Angaben der Versuchspersonen und der Reaktionszeit (Median) beziehungsweise Accuracy des Benenntests

	Alter	Geschlecht	Bildungsjahre
Alter	---		
Geschlecht	.154	---	
Bildungsjahre	.051	-.006	---
Reaktionszeit Total	.208	-.043	-.201
Reaktionszeit biologische Objekte	.279*	.042	-.194
Reaktionszeit manipulierbare Objekte	.217	.061	-.179
Accuracy Total	-.110	-.108	.317*
Accuracy biologische Objekte	-.224	-.178	.337*
Accuracy manipulierbare Objekte	.114	.058	.163

*$p < .05$, zweiseitig. **$p < .01$, zweiseitig.

Anmerkung:
Spearman’s Rho: Alter, Bildungsjahre, Reaktionszeit, Accuracy
Punktbiserale Korrelation: Geschlecht

Tabelle 3

Ergebnisse des Mann-Whitney U-Tests für die Altersgruppenunterschiede der Reaktionszeit (Median) und Accuracy des Benenntests: n unter 30 Jahre = 26, n ab 30 Jahre = 26

	Gruppe	Median bzw. Mittelwert / SD	U-Test
Reaktionszeit (RT) Total	unter 30<br>ab 30	1119<br>1292	*U* = 223, *Z* = -2.105, *p* = .035
RT biologische Objekte	unter 30<br>ab 30	1073<br>1276	*U* = 198, *Z* = -2.562, *p* = .010
RT manipulierbare Objekte	unter 30<br>ab 30	1143<br>1305	*U* = 244, *Z* = -1.720, *p* = .085
Accuracy (Acc.) Total	unter 30<br>ab 30	108.46 / 4.97<br>107.35 / 6.77	*U* = 318.5, *Z* = -.358, *p* = .720
Acc. biologische Objekte	unter 30<br>ab 30	53.77 / 3.23<br>52.23 / 5.22	*U* = 285, *Z* = -.977, *p* = .328
Acc. manipulierbare Objekte	unter 30<br>ab 30	54.69 / 2.29<br>55.12 / 2.52	*U* = 295.5, *Z* = -.788, *p* = .431

Tabelle 4

Korrelationen zwischen den Parametern des Benenntest

Parameter	1	2	3	4	5	6	7	8	9	10	11
1. Reaktionszeit (Median)	---	.372**	.005	.054	.362**	.560**	.144	.157	-.06	.194*	-.113
2. Wortfrequenz		---	.403**	.339**	.130	.079	.070	.167	.021	.337**	-.117
3. Wortlänge (Silben)			---	.622**	-.075	-.139	-.181	.056	-.080	.418**	-.241**
4. Wortlänge (Buchstaben)				---	.095	-.148	-.164	.112	-.241**	.628**	.172
5. Naming Agreement					---	.037	-.102	.094	-.263**	.210*	.079
6. Bildübereinstimmung						---	.230*	.079	.219*	-.062	-.010
7. Vertrautheit							---	.539**	.411**	-.081	.006
8. Visuelle Komplexität								---	.065	.226*	.125
9. Objektklasse									---	-.180	.000
10. Wortkomplexität										---	-.052
11. Initialfrikativ											---

*p < .05, zweiseitig. **p < .01, zweiseitig.

Anmerkung:
Spearman's Rho: Reaktionszeit, Wortfrequenz, Wortlänge (Silben), Wortlänge (Buchstaben), Naming Agreement, Bildübereinstimmung, Vertrautheit, Visuelle Komplexität

Punktbiserale Korrelation: Objektklasse, Wortkomplexität, Initialfrikativ

Tabelle 5

Ergebnisse des Mann-Whitney U-Tests für die Unterschiede der Parameter der beiden Objektklassen des Benenntests.

Parameter	Objektklasse	Mittelwert/SD	*U*-Test
Reaktionszeit (Median)	manipulierbar<br>biologisch	992<br>1033	U = 1660, Z = -.121, p = .903
Wortfrequenz	manipulierbar<br>biologisch	14.19 / 2.26<br>14.28 / 1.79	U = 1560, Z = -.683, p = .495
Wortlänge (Silben)	manipulierbar<br>biologisch	2.10 / .77<br>1.98 / .76	U = 1570, Z = -.685, p = .493
Wortlänge (Buchstaben)	manipulierbar<br>biologisch	6.90 / 2.27<br>5.91 / 1.69	U = 1269.5, Z = -2.313, p = .021
Naming Agreement	manipulierbar<br>biologisch	2.5 / 1.80<br>1.69 / 1.13	U = 1122, Z = -3.288, p < .001
Bildübereinstimmung	manipulierbar<br>biologisch	1.33 / 0.63<br>1.46 / .30	U = 1079.5, Z = -3.329, p < .001
Vertrautheit	manipulierbar<br>biologisch	1.89 / .63<br>2.50 / .72	U = 907.5, Z = -4.277, p < .001
Visuelle Komplexität	manipulierbar<br>biologisch	2.97 / .80<br>3.07 / .69	U = 1557, Z = -.690, p = .490
Wortkomplexität	manipulierbar<br>biologisch	.19 / .40<br>.07 / .26	U = 1479, Z = -1.929, p = .054
Initialfrikativ	manipulierbar<br>biologisch	.26 / .44<br>.26 / .44	U = 3393, Z = .000, p = 1.000

Tabelle 6

Resultate der Wilcoxon und McNemar χ^2 Tests der finalen Paralleltestversionen des Benenntests

Parameter	Wilcoxon *Z*-Wert	Signifikanzniveau
Summenscore	-.035	.972
Reaktionszeit	-.074	.941
Wortfrequenz	-.436	.663
Naming Agreement	-.822	.411
Bildübereinstimmung	-.024	.980

Parameter	McNemar χ^2	Signifikanzniveau
Objektklasse	.000	1.000
Wortkomplexität	.000	1.000

Tabelle 7

Innere Konsistenzwerte für die Paralleltestversionen des Benenntests

Testversion	Crombachs α
Paralleltestversion 1	.998
Paralleltestversion 2	.998

Tabelle 8

Benenntest: Paralleltestversion 1

Daten über alle Parameter der Items angeordnet anhand des aufsteigenden Summenscore

Dominante Antwort	Reaktionszeit Median (ms)	Schwierig-keitsindex	Objekt-klasse	Wort-frequenz	Silben	Buch-staben	Wort-komplexität	Frikation	Naming Agreement	Bildüber-einstimmung	Vertraut-heit	Visuelle Komplexität	Summen-score
Schere	647	0	1	13	2	6	0	1	1	1.00	1.18	2.51	675.19
Glas	676	0	1	10	1	4	0	0	2	1.17	1.10	1.75	698.02
Kerze	693	0	1	13	2	5	0	0	1	1.12	1.33	2.33	719.78
Schlange	698	0	2	11	2	8	0	1	1	1.29	3.31	4.23	731.33
Frosch	708	2	2	14	1	6	0	1	2	1.49	2.67	2.35	742.51
Krokodil	715	4	2	14	3	8	0	0	1	1.29	3.63	4.00	755.92
Banane	725	0	2	15	3	6	0	0	1	1.17	1.25	2.23	756.15
Rucksack	731	2	1	12	2	8	1	0	1	1.29	1.48	3.67	764.44
Löwe	740	0	2	13	2	4	0	0	1	1.13	3.06	3.35	769.54
Gabel	744	0	1	14	2	5	0	0	1	1.10	1.02	2.00	770.62
Hase	750	0	2	13	2	4	0	0	2	1.29	2.35	2.75	779.39
Schmetterling	764	0	2	13	3	13	0	1	2	1.15	2.21	3.90	804.76
Gürtel	777	0	1	13	2	6	0	0	2	1.23	1.15	2.79	806.17
Schaf	783	0	2	13	1	5	0	1	1	1.29	2.38	2.37	812.04
Palme	785	0	2	13	2	5	0	0	1	1.13	2.52	3.10	814.75
Zitrone	801	2	2	15	3	7	0	1	1	1.33	1.62	1.62	836.57
Trommel	804	2	1	14	2	7	0	0	1	1.13	2.47	2.78	837.43
Zebra	832	0	2	15	2	5	0	1	1	1.03	3.00	4.44	866.52
Harfe	868	0	1	15	2	5	0	0	1	1.23	3.31	4.42	900.96
Seil	916	6	1	13	1	4	0	1	3	1.35	2.29	3.49	952.13
Eule	923	0	2	16	2	4	0	0	2	1.31	3.23	3.77	956.81
Trichter	932	6	1	16	2	8	0	0	1	1.23	1.75	1.55	970.53
Erdbeere	936	0	2	16	3	8	1	0	1	1.23	1.44	3.04	972.71
Schwamm	960	2	1	14	1	7	0	1	2	1.37	1.98	4.04	995.39
Schloss	964	4	1	10	1	7	0	1	3	1.13	1.94	2.47	996.54
Bleistift	964	0	1	14	2	9	0	0	3	1.23	1.21	2.62	997.56
Huhn	968	4	2	13	1	4	0	0	2	1.52	2.37	2.94	1000.33
Giesskanne	971	2	1	15	3	10	1	0	3	1.12	1.63	2.58	1011.33
Gewehr	1057	8	1	13	2	6	0	0	3	1.33	3.27	3.88	1098.48

Dominante Antwort	Reaktionszeit Median (ms)	Schwierig-keitsindex	Objekt-klasse	Wort-frequenz	Silben	Buch-staben	Wort-komplexität	Frikation	Naming Agreement	Bildüber-einstimmung	Vertraut-heit	Visuelle Komplexität	Summen-score
Pudel	1058	8	2	15	2	5	0	0	2	1.40	2.79	2.73	1098.92
Bürste	1060	6	1	16	2	6	0	0	2	1.51	1.51	3.18	1099.20
Reh	1069	2	2	14	1	3	0	0	1	1.60	2.90	2.86	1099.36
Raupe	1061	19	2	16	2	5	0	0	1	1.63	2.86	4.20	1114.19
Mütze	1100	4	1	13	2	5	0	0	4	1.25	1.67	3.57	1135.49
Specht	1099	12	2	15	1	6	0	1	2	1.35	3.15	3.79	1146.29
Kartoffel	1110	2	2	14	3	9	0	0	1	1.46	1.46	2.81	1146.73
Flöte	1137	4	1	14	2	5	0	1	2	1.44	2.87	3.23	1173.54
Apfel	1137	10	2	13	2	5	0	0	1	1.69	1.38	1.88	1174.95
Zwiebel	1130	15	2	14	2	7	0	1	1	2.00	1.62	3.10	1178.72
Pfau	1151	23	2	15	1	4	0	0	2	1.25	3.19	4.48	1206.92
Schlittschuhe	1226	0	1	16	2	13	1	1	3	1.22	2.55	4.16	1270.93
Peperoni	1235	12	2	17	4	8	0	0	2	1.96	2.00	2.27	1286.23
Pinzette	1253	2	1	16	3	8	0	0	1	1.22	1.71	3.41	1290.34
Storch	1269	8	2	14	1	6	0	1	3	1.41	2.86	3.33	1311.10
Bügeleisen	1275	2	1	15	4	10	1	0	2	1.40	2.10	3.42	1316.92
Erbse	1283	8	2	16	2	5	0	0	5	1.69	1.98	3.06	1327.23
Beil	1291	10	1	14	1	4	0	0	2	1.13	2.49	2.47	1329.09
Pelikan	1306	23	2	17	3	7	0	0	1	1.48	3.58	3.06	1366.62
Fuchs	1323	15	2	11	1	5	0	1	1	1.65	3.12	3.39	1367.16
Hobel	1335	27	1	19	2	5	0	0	1	1.94	3.29	2.92	1398.15
Korkenzieher	1512	23	1	17	4	12	1	0	3	1.31	1.67	4.18	1579.66
Haken	1574	19	1	12	2	5	0	0	3	1.65	2.46	2.47	1622.08
Kreide	1626	27	1	14	2	6	0	0	1	2.35	2.76	3.02	1684.63
Besen	1679	8	1	14	2	5	0	0	4	2.10	1.81	3.69	1720.60
Bostitch	1704	10	1	17	2	8	0	0	4	1.27	1.54	3.69	1752.50
Klammer	1830	15	1	15	2	7	0	0	5	1.08	1.87	2.77	1880.72
Lauch	1900	48	2	17	1	5	0	0	2	2.77	2.35	3.33	1983.45
Nilpferd	1951	27	2	16	2	8	1	0	2	1.86	3.78	4.08	2018.72

Anmerkung: Da die Wortfrequenz für das Wort „Bostitch“ nicht zur Verfügung stand, wurde die Frequenz des Synonyms „Hefter“ verwendet.

Tabelle 9

Benenntest: Paralleltestversion 2

Daten über alle Parameter der Items angeordnet anhand des aufsteigenden Summenscores

Dominante Antwort	Reaktionszeit Median (ms)	Schwierig-keitsindex	Objekt-klasse	Wort-frequenz	Silben	Buch-staben	Wort-komplexität	Frikation	Naming Agreement	Bildüber-einstimmung	Vertraut-heit	Visuelle Komplexität	Summen-score
Hut	611	0	1	11	1	3	0	0	1	1.10	1.90	2.31	633.31
Schnecke	662	2	2	15	2	8	0	1	2	1.10	1.98	2.94	700.02
Schlüssel	675	0	1	11	2	9	0	1	1	1.21	1.06	2.23	704.00
Löffel	678	0	1	14	2	6	0	0	2	1.08	1.00	1.58	706.66
Hammer	701	0	1	12	2	6	0	0	1	1.12	1.63	1.94	727.19
Elefant	702	0	2	14	2	7	0	0	1	1.06	2.90	2.69	734.65
Koffer	718	8	1	11	2	6	0	0	2	1.46	1.65	2.85	753.96
Schwan	726	8	2	13	1	6	0	1	1	1.48	2.37	2.75	764.60
Gitarre	730	4	1	12	3	7	0	0	1	1.17	2.56	3.65	765.38
Tasse	760	0	1	13	2	5	0	0	3	1.10	1.10	1.78	787.98
Kamm	775	0	1	14	1	4	0	0	3	1.10	1.67	3.17	803.44
Pinguin	773	0	2	15	3	7	0	0	1	1.15	3.08	3.27	808.00
Staubsauger	779	0	1	14	3	11	1	1	1	1.08	1.35	4.18	817.11
Klavier	789	2	1	12	2	7	0	0	2	1.37	2.15	3.31	821.83
Lampe	805	0	1	13	2	5	0	0	4	1.29	1.25	2.20	834.24
Rollschuh	809	0	1	18	2	10	1	0	2	1.21	2.54	4.19	850.94
Messer	838	0	1	11	2	6	0	0	1	1.12	1.04	1.54	862.20
Känguruh	865	0	2	18	3	8	0	0	1	1.13	3.44	3.12	904.19
Dackel	881	2	2	15	2	6	0	0	2	1.29	2.88	2.61	916.78
Birne	888	0	2	15	2	5	0	0	1	1.38	1.42	2.08	917.88
Spinne	880	13	2	14	2	6	0	1	1	1.46	2.23	3.94	926.63
Katze	929	0	2	11	2	5	0	0	1	1.37	1.54	2.56	954.97
Kette	937	0	1	12	2	5	0	0	4	1.44	2.04	2.35	966.33
Kaktus	939	2	2	16	2	6	0	0	2	1.29	2.96	3.35	976.60
Nashorn	954	4	2	16	2	7	1	0	2	1.27	3.48	3.42	996.17
Esel	969	4	2	14	1	4	0	0	1	1.18	2.55	3.25	1001.48
Libelle	952	17	2	17	3	7	0	0	1	1.37	3.00	2.87	1006.24
Tomate	974	2	2	15	3	6	0	0	1	1.62	1.31	2.06	1007.99
Pistole	976	4	1	12	3	7	0	0	3	1.19	3.38	4.00	1014.57

Dominante Antwort	Reaktionszeit Median (ms)	Schwierig-keitsindex	Objekt-klasse	Wort-frequenz	Silben	Buch-staben	Wort-komplexität	Frikation	Naming Agreement	Bildüber-einstimmung	Vertraut-heit	Visuelle Komplexität	Summen-score
Sieb	1008	6	1	15	1	4	0	1	1	1.63	1.65	2.85	1043.13
Karotte	1009	2	2	17	3	7	0	0	5	1.25	1.33	2.40	1049.98
Zange	1033	4	1	15	2	5	0	1	3	1.21	2.19	2.73	1069.63
Schraube	1084	2	1	14	2	8	0	1	2	1.10	1.76	3.72	1120.58
Wolf	1134	6	2	10	1	4	0	0	2	1.60	3.71	4.35	1168.66
Wurm	1154	4	2	13	1	4	0	0	2	1.54	2.38	3.27	1187.19
Trompete	1156	4	1	14	3	8	0	0	1	1.33	2.86	4.24	1194.93
Hirsch	1146	17	2	13	1	6	0	0	3	1.52	3.12	2.62	1195.26
Spargel	1159	12	2	13	2	7	0	1	1	1.45	1.96	2.71	1202.62
Radiergummi	1150	12	1	17	3	11	1	0	2	1.35	1.51	3.06	1202.92
Ente	1177	0	2	13	2	4	0	0	1	1.60	2.31	2.67	1205.08
Melone	1168	12	2	16	3	6	0	0	2	1.55	1.80	3.75	1216.10
Ziege	1190	17	2	14	2	5	0	1	3	1.42	2.75	3.44	1241.61
Tiger	1258	15	2	11	2	5	0	0	1	1.77	3.37	3.84	1302.48
Erdnuss	1279	10	2	14	2	7	1	0	7	1.38	1.79	2.63	1327.80
Pinsel	1301	4	1	14	2	6	0	0	1	1.21	2.13	2.46	1334.80
Gurke	1295	13	2	15	2	5	0	0	1	1.96	1.65	2.80	1339.41
Schnur	1367	2	1	14	1	6	0	1	11	1.63	1.83	3.67	1410.13
Gans	1428	33	2	14	1	4	0	0	1	1.69	3.04	2.47	1490.20
Schaufel	1478	4	1	14	2	8	0	1	2	1.62	1.63	2.18	1515.43
Raffel	1511	13	1	20	2	6	0	0	6	1.29	1.73	3.56	1565.58
Füllfeder	1521	0	1	20	3	9	1	1	8	1.58	2.37	3.85	1571.80
Hahn	1538	10	2	11	1	4	0	0	2	1.29	2.50	3.50	1575.29
Büroklammer	1528	10	1	17	4	11	1	0	4	1.00	1.19	2.13	1580.32
Orange	1536	19	2	12	2	6	0	0	2	1.76	1.35	1.73	1583.84
Kürbis	1572	35	2	16	2	6	0	0	1	2.10	2.56	2.42	1640.58
Schlauch	1636	27	1	14	1	8	0	1	3	2.27	2.21	1.69	1697.17
Locher	1951	8	1	17	2	6	0	0	2	1.08	1.31	2.63	1992.02
Dosenöffner	1995	12	1	17	4	11	1	0	3	1.94	1.73	3.23	2050.40

Anhang A

Benenntest Paralleltestversion 1

Anmerkung: Bilder sind nach aufsteigender Schwierigkeit geordnet von links nach rechts.

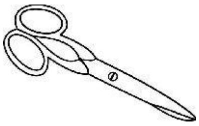
Schere

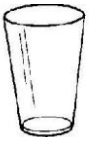
Glas

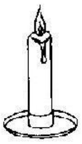
Kerze

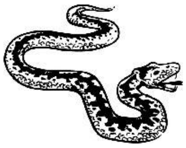
Schlange

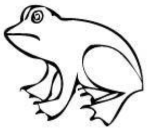
Frosch

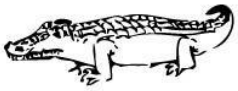
Krokodil

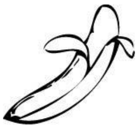
Banane

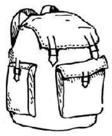
Rucksack

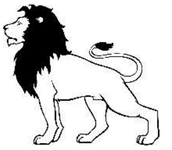
Löwe

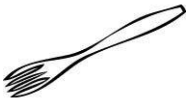
Gabel

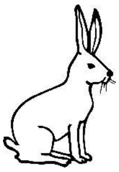
Hase

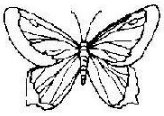
Schmetterling

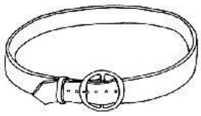
Gürtel

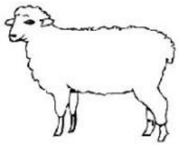
Schaf

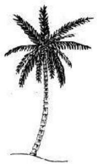
Palme

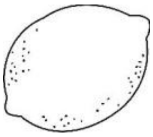
Zitrone

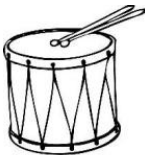
Trommel

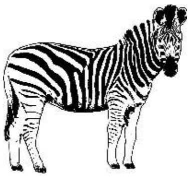
Zebra

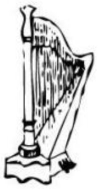
Harfe

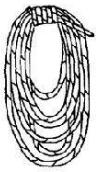
Seil

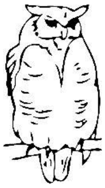
Eule

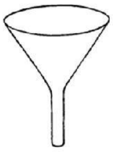
Trichter

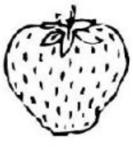
Erdbeere

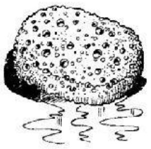
Schwamm

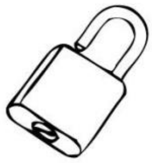
Schloss

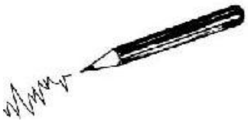
Bleistift

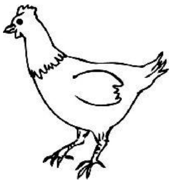
Huhn

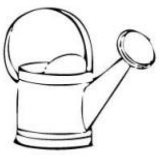
Giesskanne

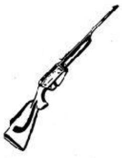
Gewehr

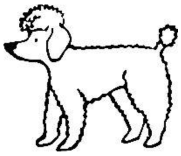
Pudel

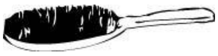
Bürste

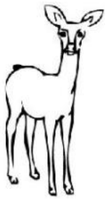
Reh

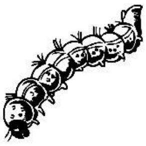
Raupe

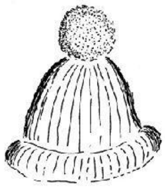
Mütze

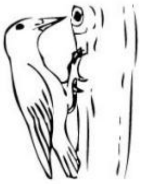
Specht

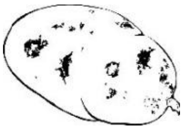
Kartoffel

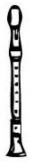
Flöte

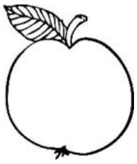
Apfel

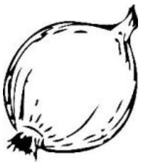
Zwiebel

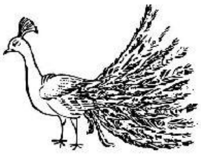
Pfau

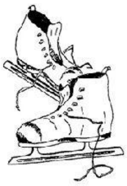
Schlittschuhe

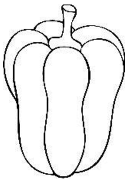
Peperoni

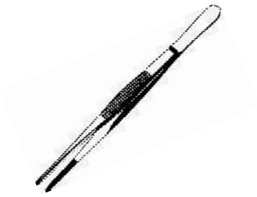
Pinzette

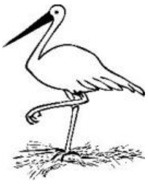
Storch

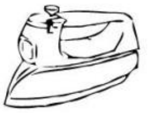
Bügeleisen

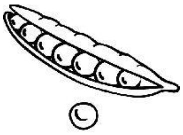
Erbse

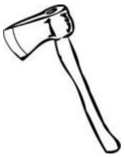
Beil

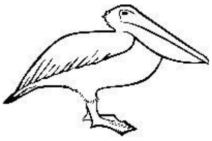
Pelikan

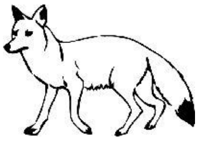
Fuchs

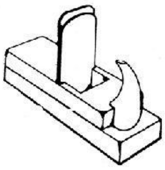
Hobel

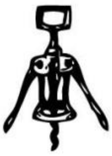
Korkenzieher

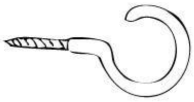
Haken

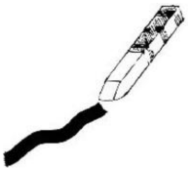
Kreide

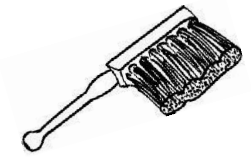
Besen

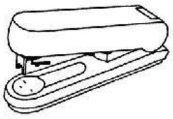
Bostitch

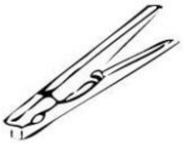
Klammer

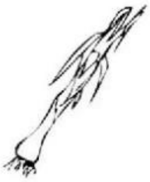
Lauch

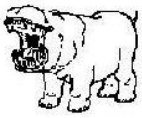
Nilpferd

Anhang B

Benenntest Parallelversion 2

Anmerkung: Bilder sind nach aufsteigender Schwierigkeit geordnet von links nach rechts.

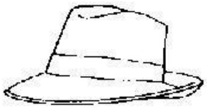
Hut

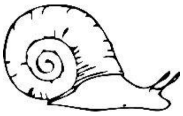
Schnecke

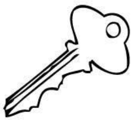
Schlüssel

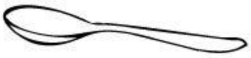
Löffel

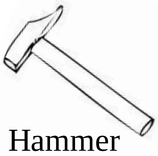
Hammer

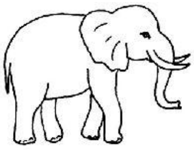
Elefant

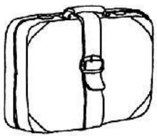
Koffer

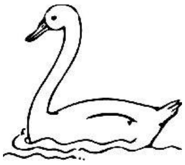
Schwan

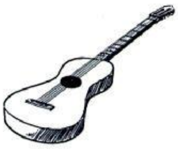
Gitarre

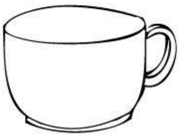
Tasse

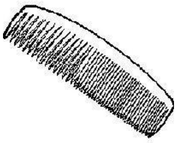
Kamm

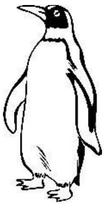
Pinguin

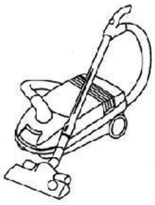
Staubsauger

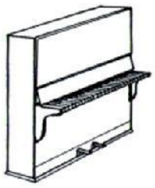
Klavier

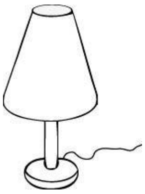
Lampe

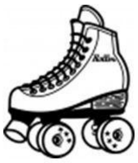
Rollschuh

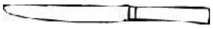
Messer

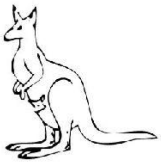
Känguru

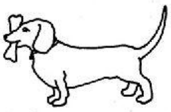
Dackel

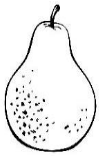
Birne

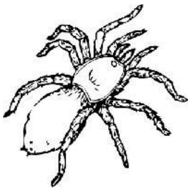
Spinne

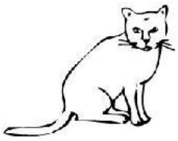
Katze

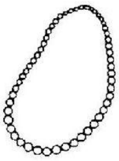
Kette

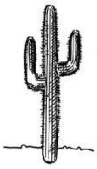
Kaktus

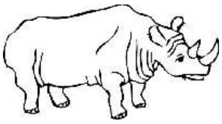
Nashorn

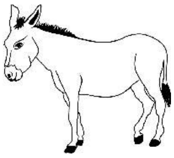
Esel

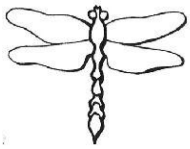
Libelle

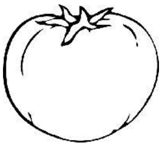
Tomate

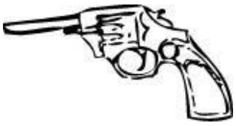
Pistole

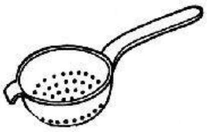
Sieb

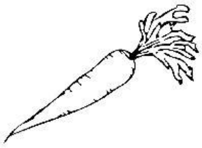
Karotte

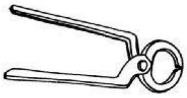
Zange

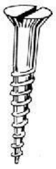
Schraube

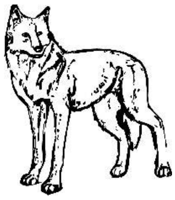
Wolf

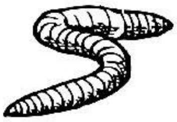
Wurm

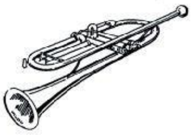
Trompete

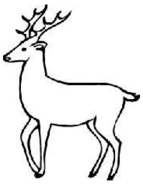
Hirsch

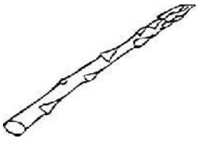
Spargel

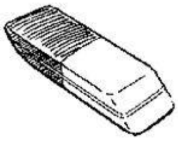
Radiergummi

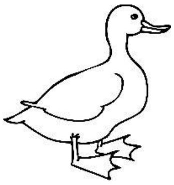
Ente

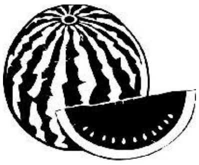
Melone

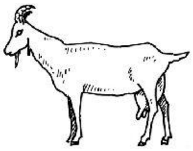
Ziege

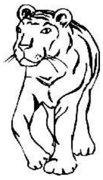
Tiger

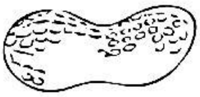
Erdnuss

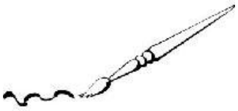
Pinsel

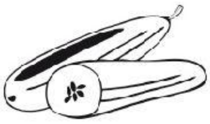
Gurke

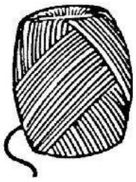
Schnur

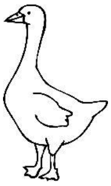
Gans

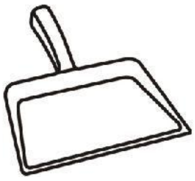
Schaufel

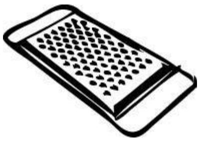
Raffel

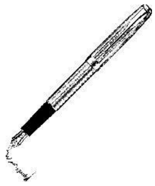
Füllfeder

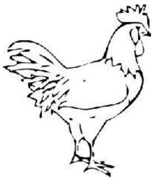
Hahn

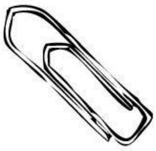
Büroklammer

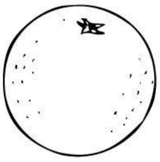
Orange

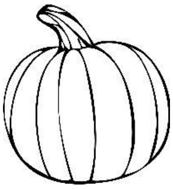
Kürbis

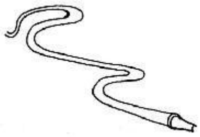
Schlauch

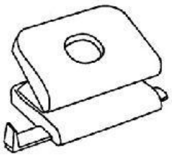
Locher

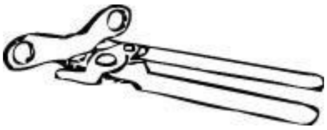
Dosenöffner

Tabelle 10

Korrelationen zwischen den demographischen Angaben der Versuchspersonen und der Reaktionszeit beziehungsweise Accuracy des semantischen Entscheidungstests

	Alter	Geschlecht	Bildungsjahre	Händigkeit
Alter	---			
Geschlecht	.154	---		
Bildungsjahre	.051	-.006	---	
Händigkeit	-.025	.073	.036	---
Reaktionszeit (Median)				
Total	.633**	.068	-.167	.269
Biologische Objekte	.638**	.114	-.166	.250
Manipulierbare Objekte	.616**	.014	-.143	.284*
Kongruente Bild-Wort-Paare	.624**	.068	-.108	.283*
Inkongruente Bild-Wort-Paare	.645**	.067	-.176	.073
Accuracy				
Total	-.004	.016	.140	-.235
Biologische Objekte	-.117	-.061	.151	-.194
Manipulierbare Objekte	.062	.082	.101	-.229
Kongruente Bild-Wort-Paare	.061	-.028	.034	-.131
Inkongruente Bild-Wort-Paare	-.044	.026	.187	-.233

*$p < .05$, zweiseitig. **$p < .01$, zweiseitig.

Anmerkung:
Spearman's Rho: Alter, Bildungsjahre, Reaktionszeit, Accuracy
Punktbiserale Korrelation: Geschlecht, Händigkeit

Tabelle 11

Ergebnisse des Mann-Whitney U-Tests für die Altersgruppenunterschiede der Reaktionszeit und Accuracy des sem. Entscheidungstest: n unter 30 Jahre = 26, n ab 30 Jahre = 26

Reaktionszeit	Gruppe	Median	*U*-Test
Total	unter 30 Jahre<br>ab 30 Jahre	1045<br>1436	$U = 111$, $Z = -4.154$, $p < .001$
Biologische Objekte	unter 30 Jahre<br>ab 30 Jahre	1004<br>1456	$U = 113$, $Z = -4.118$, $p < .001$
Manipulierbare Objekte	unter 30 Jahre<br>ab 30 Jahre	1079<br>1404	$U = 118$, $Z = -4.026$, $p < .001$
Kongruente Bild-Wort-Paare	unter 30 Jahre<br>ab 30 Jahre	992<br>1330	$U = 122$, $Z = -3.953$, $p < .001$
Inkongruente Bild-Wort-Paare	unter 30 Jahre<br>ab 30 Jahre	1104<br>1571	$U = 105$, $Z = -4.264$, $p < .001$

Accuracy	Gruppe	Mittelwert / SD	*U*-Test
Total	unter 30 Jahre<br>ab 30 Jahre	220.69 / 6.81<br>221.62 / 7.09	$U = 293$, $Z = 0.825$, $p = .409$
Biologische Objekte	unter 30 Jahre<br>ab 30 Jahre	111.31 / 3.37<br>110.96 / 3.89	$U = 324.5$, $Z = -.249$, $p = .803$
Manipulierbare Objekte	unter 30 Jahre<br>ab 30 Jahre	109.38 / 4.18<br>110.65 / 111.5	$U = 259.5$, $Z = -1.445$, $p = .148$
Kongruente Bild-Wort-Paare	unter 30 Jahre<br>ab 30 Jahre	112.88 / 1.68<br>113.5 / 1.556	$U = 266.5$, $Z = -1.337$, $p = .181$
Inkongruente Bild-Wort-Paare	unter 30 Jahre<br>ab 30 Jahre	107.81 / 5.76<br>108.12 / 6.03	$U = 308.5$, $Z = -.542$, $p = .588$

Tabelle 12

Ergebnisse des Mann-Whitney U-Tests für die Händigkeitsunterschiede der Reaktionszeit und Accuracy des semantischen Entscheidungstest: n Rechtshänder = 49, n Linkshänder = 3

Reaktionszeit	Gruppe	Median	*U*-Test
Total	rechts<br>links	1153<br>1702	$U = 24$, $Z = -1.943$, $p = .052$
Biologische Objekte	rechts<br>links	1140<br>1739	$U = 30$, $Z = -1.707$, $p = .088$
Manipulierbare Objekte	rechts<br>links	1167<br>1662	$U = 22$, $Z = -2.021$, $p = .043$
Konsistente Bild-Wort-Paare	rechts<br>links	1118<br>1618	$U = 25$, $Z = -1.903$, $p = .057$
Inkonsistente Bild-Wort-Paare	rechts<br>links	1192<br>1764	$U = 28$, $Z = -1.786$, $p = .074$

Accuracy	Gruppe	Mittelwert/SD	*U*-Test
Total	rechts<br>links	221.55 / 6.35<br>214.67 / 13.32	$U = 48{,}5$, $Z = -.983$, $p = .326$
Biologische Objekte	rechts<br>links	111.31 / 3.47<br>108.33 / 5.51	$U = 49$, $Z = -.968$, $p = .333$
Manipulierbare Objekte	rechts<br>links	110.24 / 3.68<br>106.33 / 8.15	$U = 52.5$, $Z = -.829$, $p = .407$
Konsistente Bild-Wort-Paare	rechts<br>links	113.24 / 1.55<br>112.33 / 3.06	$U = 62$, $Z = -.461$, $p = .645$
Inkonsistente Bild-Wort-Paare	rechts<br>links	108.31 / 5.67<br>102.33 / 10.26	$U = 43.5$, $Z = -1.182$, $p = .237$

Tabelle 13

Ergebnisse der zweifaktoriellen Varianzanalyse Objektklasse (biologisch, manipulierbar) x Kongruenz (kongruent, inkongruent) mit Messwiederholung auf beiden Faktoren für die Reaktionszeit der Items des Semantischen Entscheidungstests

	F-Wert	Freiheitsgrade	Fehler	Signifikanzniveau
Objektklasse	1.107	1	57	.297
Kongruenz	62.701	1	57	.001
Objektklasse x Kongruenz	14.525	1	57	.001

		Mittelwert	Standardfehler
Faktor Objektklasse	belebt	1110	14.152
	manipulierbar	1134	18.489
Faktor Kongruenz	kongruent	1053	12.816
	inkongruent	1191	16.664
Interaktion Objektklasse x Kongruenz			
Belebt	kongruent	1072	17.706
	inkongruent	1149	17.326
Manipulierbar	kongruent	1034	17.856
	inkongruent	1234	26.756

Tabelle 14

Ergebnisse der zweifaktoriellen Varianzanalyse Objektklasse (biologisch, manipulierbar) x Kongruenz (kongruent, inkongruent) mit Messwiederholung auf beiden Faktoren für die Accuracy der Items des Semantischen Entscheidungstests:

	F-Wert	Freiheitsgrade	Fehler	Signifikanzniveau
Objektklasse	1.685	1	57	.199
Kongruenz	29.456	1	57	.001
Objektklasse x Kongruenz	1.271	1	57	.264

		Mittelwert	Standardfehler
Faktor Objektklasse	belebt	49.819	.283
	manipulierbar	49.319	.321
Faktor Kongruenz	kongruent	50.741	.183
	inkongruent	48.397	.411
Interaktion Objektklasse x Kongruenz			
Belebt	kongruent	50.741	.187
	inkongruent	48.897	.521
Manipulierbar	kongruent	50.741	.339
	inkongruent	47.897	.574

Tabelle 15

Semantischer Entscheidungstest: Paralleltestversion 1

Daten über alle Parameter der Bild-Wort-Paare, welche randomisiert angeordnet sind

Item (Bild)	Item (Wort)	Kongruenz	Objektklasse	Reaktionszeit Median (ms)	Schwierigkeitsindex
Peperoni	Kürbis	inkongruent	biologisch	1319	92
Palme	Kaktus	Inkongruent	biologisch	1072	100
Bürste	Kamm	inkongruent	manipulierbar	1268	85
Beil	Beil	kongruent	manipulierbar	1178	96
Frosch	Känguru	inkongruent	biologisch	1118	98
Besen	Schaufel	inkongruent	manipulierbar	1140	100
Pelikan	Pinguin	inkongruent	biologisch	1209	96
Krokodil	Krokodil	kongruent	biologisch	971	100
Huhn	Gans	inkongruent	biologisch	1180	96
Mütze	Hut	inkongruent	manipulierbar	1381	73
Schlittschuhe	Schlittschuhe	kongruent	manipulierbar	1209	96
Haken	Haken	kongruent	manipulierbar	1218	92
Bügeleisen	Bügeleisen	kongruent	manipulierbar	962	98
Lauch	Lauch	kongruent	biologisch	1323	90
Kerze	Lampe	inkongruent	manipulierbar	1140	94
Bleistift	Pinsel	inkongruent	manipulierbar	1705	92
Schlange	Schnecke	inkongruent	biologisch	1245	100
Gabel	Gabel	kongruent	manipulierbar	970.5	100
Pudel	Pudel	kongruent	biologisch	1057	98
Reh	Reh	kongruent	biologisch	1088	100
Zwiebel	Tomate	inkongruent	biologisch	1191	100
Trichter	Trichter	kongruent	manipulierbar	1028	100
Rucksack	Rucksack	kongruent	manipulierbar	870	98
Hobel	Raffel	inkongruent	manipulierbar	1717	63
Hase	Hase	kongruent	biologisch	1052	98
Giesskanne	Giesskanne	kongruent	manipulierbar	917	100
Fuchs	Wolf	inkongruent	biologisch	1411	62
Erbse	Gurke	inkongruent	biologisch	938	100
Raupe	Raupe	kongruent	biologisch	1117	90
Zitrone	Melone	inkongruent	biologisch	1145	96
Schmetterling	Schmetterling	kongruent	biologisch	985	98
Schere	Messer	inkongruent	manipulierbar	947	96
Löwe	Tiger	inkongruent	biologisch	1066	92
Pfau	Hahn	inkongruent	biologisch	1119	100
Apfel	Apfel	kongruent	biologisch	1136	96
Gewehr	Pistole	inkongruent	manipulierbar	1526	85
Schloss	Schloss	kongruent	manipulierbar	907	98

Item (Bild)	Item (Wort)	Kongruenz	Objektklasse	Reaktionszeit Median (ms)	Schwierigkeitsindex
Pelikan	Pelikan	kongruent	biologisch	953	94
Bügeleisen	Staubsauger	inkongruent	manipulierbar	947	100
Schwamm	Schwamm	kongruent	manipulierbar	959	100
Gabel	Löffel	inkongruent	manipulierbar	1153	98
Nilpferd	Nashorn	inkongruent	biologisch	1426	90
Hase	Katze	inkongruent	biologisch	1007	98
Erdbeere	Erdbeere	kongruent	biologisch	1000	96
Glas	Glas	kongruent	manipulierbar	1026	98
Trommel	Klavier	inkongruent	manipulierbar	962	96
Mütze	Mütze	kongruent	manipulierbar	885	98
Seil	Seil	kongruent	manipulierbar	862	98
Kartoffel	Kartoffel	kongruent	biologisch	1017	98
Korkenzieher	Dosenöffner	inkongruent	manipulierbar	1627	85
Zebra	Esel	inkongruent	biologisch	1178	94
Bleistift	Bleistift	kongruent	manipulierbar	1003	100
Klammer	Büroklammer	inkongruent	manipulierbar	1473	96
Banane	Banane	kongruent	biologisch	804	100
Bostitch	Bostitch	kongruent	manipulierbar	1411	92
Storch	Schwan	inkongruent	biologisch	1023	96
Schaf	Schaf	kongruent	biologisch	1161	98
Apfel	Birne	inkongruent	biologisch	1053	96
Specht	Spinne	inkongruent	biologisch	1099	100
Haken	Schraube	inkongruent	manipulierbar	1323	75
Giesskanne	Schlauch	inkongruent	manipulierbar	1141	98
Harfe	Harfe	kongruent	manipulierbar	1008	96
Gewehr	Gewehr	kongruent	manipulierbar	1145	100
Schlange	Schlange	kongruent	biologisch	881	100
Flöte	Trompete	inkongruent	manipulierbar	1302	90
Beil	Hammer	inkongruent	manipulierbar	1263	92
Zitrone	Zitrone	kongruent	biologisch	943	100
Schwamm	Radiergummi	inkongruent	manipulierbar	1037	98
Pinzette	Zange	inkongruent	manipulierbar	1072	94
Huhn	Huhn	kongruent	biologisch	1376	96
Eule	Ente	inkongruent	biologisch	1153	100
Reh	Hirsch	inkongruent	biologisch	1150	94
Peperoni	Peperoni	kongruent	biologisch	1238	88
Schere	Schere	kongruent	manipulierbar	1018	100
Gürtel	Gürtel	kongruent	manipulierbar	868	96
Frosch	Frosch	kongruent	biologisch	960	98
Lauch	Spargel	inkongruent	biologisch	1340	98
Banane	Orange	inkongruent	biologisch	903	96
Schmetterling	Libelle	inkongruent	biologisch	1112	87
Seil	Schnur	inkongruent	manipulierbar	1422	75

Item (Bild)	Item (Wort)	Kongruenz	Objektklasse	Reaktionszeit Median (ms)	Schwierigkeitsindex
Krokodil	Elefant	inkongruent	biologisch	985	96
Zwiebel	Zwiebel	kongruent	biologisch	1256	92
Besen	Besen	kongruent	manipulierbar	1186	65
Raupe	Wurm	inkongruent	biologisch	1337	87
Kerze	Kerze	kongruent	manipulierbar	811	100
Pudel	Dackel	inkongruent	biologisch	1247	88
Hobel	Hobel	kongruent	manipulierbar	1011	98
Bostitch	Locher	inkongruent	manipulierbar	1512	79
Korkenzieher	Korkenzieher	kongruent	manipulierbar	1106	98
Erdbeere	Erdnuss	inkongruent	biologisch	1037	96
Zebra	Zebra	kongruent	biologisch	1073	98
Schaf	Ziege	inkongruent	biologisch	1231	94
Eule	Eule	kongruent	biologisch	850	100
Schloss	Schlüssel	inkongruent	manipulierbar	1132	94
Harfe	Gitarre	inkongruent	manipulierbar	1119	100
Nilpferd	Nilpferd	kongruent	biologisch	1393	94
Kreide	Kreide	kongruent	manipulierbar	1281	88
Glas	Tasse	inkongruent	manipulierbar	1009	96
Storch	Storch	kongruent	biologisch	1161	98
Pfau	Pfau	kongruent	biologisch	1117	96
Palme	Palme	kongruent	biologisch	877	98
Trichter	Sieb	inkongruent	manipulierbar	1454	88
Erbse	Erbse	kongruent	biologisch	942	98
Trommel	Trommel	kongruent	manipulierbar	840	98
Bürste	Bürste	kongruent	manipulierbar	1092	98
Rucksack	Koffer	inkongruent	manipulierbar	1191	96
Klammer	Klammer	kongruent	manipulierbar	1198	94
Gürtel	Kette	inkongruent	manipulierbar	1124	98
Flöte	Flöte	kongruent	manipulierbar	945	100
Kartoffel	Karotte	inkongruent	biologisch	1308	90
Pinzette	Pinzette	kongruent	manipulierbar	1089	98
Schlittschuhe	Rollschuhe	inkongruent	manipulierbar	1381	94
Löwe	Löwe	kongruent	biologisch	974	100
Specht	Specht	kongruent	biologisch	1095	98
Fuchs	Fuchs	kongruent	biologisch	1076	100
Kreide	Füllfeder	inkongruent	manipulierbar	1300	96

Tabelle 16

Semantischer Entscheidungstest: Paralleltestversion 2

Daten über alle Parameter der Bild-Wort-Paare, welche randomisiert angeordnet sind

Item (Bild)	Item (Wort)	Kongruenz	Objektklasse	Reaktionszeit Median (ms)	Schwierigkeitsindex
Melone	Melone	kongruent	biologisch	1231	98
Esel	Zebra	inkongruent	biologisch	1177	100
Wolf	Fuchs	inkongruent	biologisch	1321	88
Känguru	Känguru	kongruent	biologisch	1051	98
Orange	Banane	inkongruent	biologisch	1117	98
Dackel	Dackel	kongruent	biologisch	1044	92
Kamm	Bürste	inkongruent	manipulierbar	1412	77
Schwan	Schwan	kongruent	biologisch	1045	96
Locher	Locher	kongruent	manipulierbar	1065	100
Pistole	Gewehr	inkongruent	manipulierbar	1108	92
Pinsel	Bleistift	inkongruent	manipulierbar	1318	98
Schaufel	Besen	inkongruent	manipulierbar	1040	100
Hut	Hut	kongruent	manipulierbar	924	98
Löffel	Gabel	inkongruent	manipulierbar	1002	96
Birne	Apfel	inkongruent	biologisch	1090	100
Schnecke	Schnecke	kongruent	biologisch	919	100
Staubsauger	Staubsauger	kongruent	manipulierbar	988	100
Gans	Huhn	inkongruent	biologisch	1043	100
Gurke	Erbse	inkongruent	biologisch	1190	100
Rollschuh	Rollschuh	kongruent	manipulierbar	1216	98
Tasse	Tasse	kongruent	manipulierbar	962	98
Nashorn	Nilpferd	inkongruent	biologisch	1320	79
Tiger	Tiger	kongruent	biologisch	1029	100
Trompete	Flöte	inkongruent	manipulierbar	1105	96
Hahn	Hahn	kongruent	biologisch	1226	98
Pistole	Pistole	kongruent	manipulierbar	1102	100
Kürbis	Kürbis	kongruent	biologisch	1270	94
Karotte	Kartoffel	inkongruent	biologisch	1251	94
Zange	Pinzette	inkongruent	manipulierbar	1062	96
Koffer	Koffer	kongruent	manipulierbar	878	100
Schraube	Haken	inkongruent	manipulierbar	1209	94
Ziege	Ziege	kongruent	biologisch	1106	96
Schlüssel	Schloss	inkongruent	manipulierbar	1008	98
Kaktus	Palme	inkongruent	biologisch	1073	100
Hammer	Beil	inkongruent	manipulierbar	1178	96
Pinguin	Pinguin	kongruent	biologisch	811	100
Rollschuh	Schlittschuh	inkongruent	manipulierbar	1144	96

Item (Bild)	Item (Wort)	Kongruenz	Objektklasse	Reaktionszeit Median (ms)	Schwierigkeitsindex
Gans	Gans	kongruent	biologisch	1117	100
Klavier	Trommel	inkongruent	manipulierbar	1081	98
Tomate	Zwiebel	inkongruent	biologisch	1233	92
Wolf	Wolf	kongruent	biologisch	1059	100
Katze	Katze	kongruent	biologisch	1031	100
Raffel	Raffel	kongruent	manipulierbar	1057	100
Dosenöffner	Dosenöffner	kongruent	manipulierbar	1297	98
Schlauch	Giesskanne	inkongruent	manipulierbar	1209	100
Radiergummi	Radiergummi	kongruent	manipulierbar	968	100
Hirsch	Reh	inkongruent	biologisch	1157	75
Zange	Zange	kongruent	manipulierbar	983	96
Schnur	Seil	inkongruent	manipulierbar	1557	90
Wurm	Raupe	inkongruent	biologisch	1210	85
Hahn	Pfau	inkongruent	biologisch	1061	98
Füllfeder	Füllfeder	kongruent	manipulierbar	1281	100
Känguru	Frosch	inkongruent	biologisch	964	100
Schnecke	Schlange	inkongruent	biologisch	1013	96
Kette	Gürtel	inkongruent	manipulierbar	972	98
Orange	Orange	kongruent	biologisch	1146	98
Gurke	Gurke	kongruent	biologisch	1219	98
Elefant	Krokodil	inkongruent	biologisch	952	100
Libelle	Schmetterling	inkongruent	biologisch	1154	92
Büroklammer	Klammer	inkongruent	manipulierbar	1372	90
Ente	Huhn	inkongruent	biologisch	963	98
Nashorn	Nashorn	kongruent	biologisch	1265	98
Klavier	Klavier	kongruent	manipulierbar	1041	98
Schraube	Schraube	kongruent	manipulierbar	1033	100
Schwan	Storch	inkongruent	biologisch	968	100
Erdnuss	Erdbeere	inkongruent	biologisch	1216	96
Spargel	Spargel	kongruent	biologisch	1108	98
Lampe	Kerze	inkongruent	manipulierbar	946	96
Dosenöffner	Korkenzieher	inkongruent	manipulierbar	1272	96
Ziege	Schaf	inkongruent	biologisch	1060	98
Sieb	Sieb	kongruent	manipulierbar	1079	98
Spinne	Specht	inkongruent	biologisch	1261	100
Messer	Messer	kongruent	manipulierbar	1010	100
Schnur	Schnur	kongruent	manipulierbar	1129	98
Kaktus	Kaktus	kongruent	biologisch	1091	98
Tasse	Glas	inkongruent	manipulierbar	1183	94
Birne	Birne	kongruent	biologisch	1073	100
Hut	Mütze	inkongruent	manipulierbar	1207	77
Wurm	Wurm	kongruent	biologisch	1033	94
Karotte	Karotte	kongruent	biologisch	966	98

Item (Bild)	Item (Wort)	Kongruenz	Objektklasse	Reaktionszeit Median (ms)	Schwierigkeitsindex
Trompete	Trompete	kongruent	manipulierbar	984	100
Libelle	Libelle	kongruent	biologisch	1135	100
Pinguin	Pelikan	inkongruent	biologisch	1039	90
Kette	Kette	kongruent	manipulierbar	1023	100
Gitarre	Gitarre	kongruent	manipulierbar	843	98
Melone	Zitrone	inkongruent	biologisch	1070	100
Hirsch	Hirsch	kongruent	biologisch	1150	98
Raffel	Hobel	inkongruent	manipulierbar	1267	81
Radiergummi	Schwamm	inkongruent	manipulierbar	1324	88
Schlauch	Schlauch	kongruent	manipulierbar	1329	100
Spargel	Lauch	inkongruent	biologisch	1343	90
Pinsel	Pinsel	kongruent	manipulierbar	1086	98
Lampe	Lampe	kongruent	manipulierbar	926	100
Löffel	Löffel	kongruent	manipulierbar	887	96
Schaufel	Schaufel	kongruent	manipulierbar	1137	90
Tomate	Tomate	kongruent	biologisch	1274	100
Esel	Esel	kongruent	biologisch	1040	100
Ente	Ente	kongruent	biologisch	1126	96
Locher	Bostitch	inkongruent	manipulierbar	1567	79
Dackel	Pudel	inkongruent	biologisch	1477	75
Gitarre	Harfe	inkongruent	manipulierbar	1196	100
Büroklammer	Büroklammer	kongruent	manipulierbar	997	98
Sieb	Trichter	inkongruent	manipulierbar	1570	88
Elefant	Elefant	kongruent	biologisch	951	100
Katze	Hase	inkongruent	biologisch	1171	98
Füllfeder	Kreide	inkongruent	manipulierbar	1427	100
Kamm	Kamm	kongruent	manipulierbar	941	100
Staubsauger	Bügeleisen	inkongruent	manipulierbar	1036	98
Tiger	Löwe	inkongruent	biologisch	1127	81
Kürbis	Melone	inkongruent	biologisch	1010	92
Hammer	Hammer	kongruent	manipulierbar	929	100
Schlüssel	Schlüssel	kongruent	manipulierbar	898	100
Messer	Schere	inkongruent	manipulierbar	998	100
Koffer	Rucksack	inkongruent	manipulierbar	1030	98
Erdnuss	Erdnuss	kongruent	biologisch	863	100
Spinne	Spinne	kongruent	biologisch	935	96

Anhang C

Semantischer Entscheidungstest Paralleltestversion 1

Anmerkung: Bild-Wort-Paare sind randomisiert angeordnet

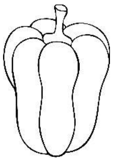
Kürbis

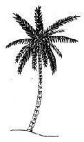
Kaktus

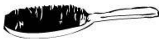
Kamm

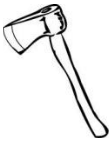
Beil

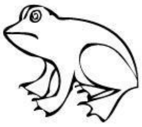
Känguru

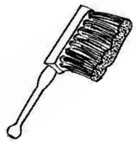
Schaufel

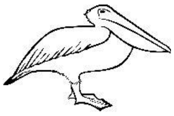
Pinguin

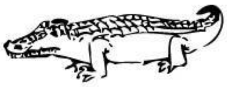
Krokodil

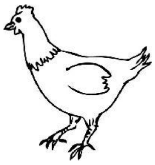
Gans

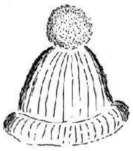
Hut

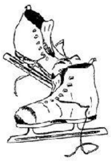
Schlittschuhe

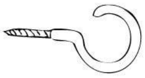
Haken

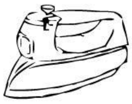
Bügeleisen

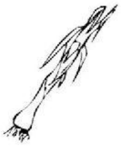
Lauch

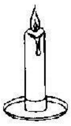
Lampe

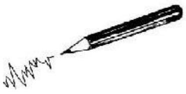
Pinsel

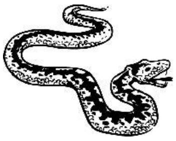
Schnecke

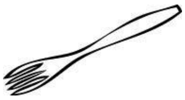
Gabel

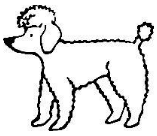
Pudel

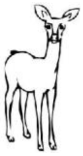
Reh

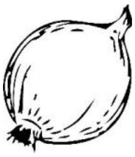
Tomate

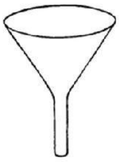
Trichter

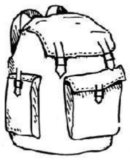
Rucksack

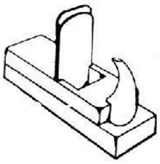
Raffel

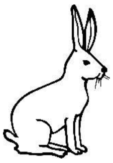
Hase

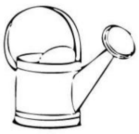
Giesskanne

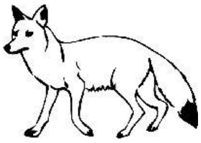
Wolf

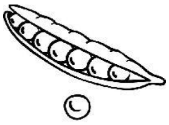
Gurke

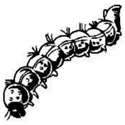
Raupe

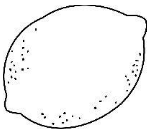
Melone

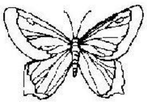
Schmetterling

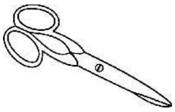
Messer

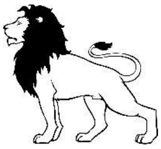
Tiger

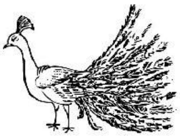
Hahn

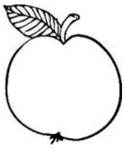
Apfel

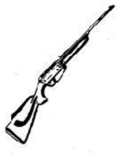 Pistole	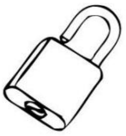 Schloss	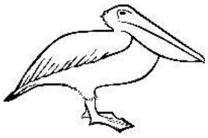 Pelikan	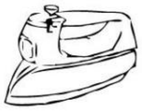 Staubsauger	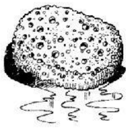 Schwamm
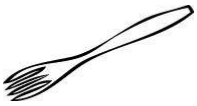 Löffel	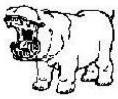 Nashorn	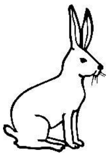 Katze	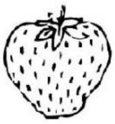 Erdbeere	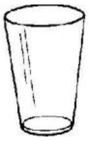 Glas
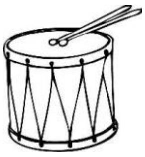 Klavier	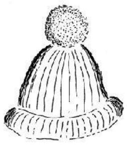 Mütze	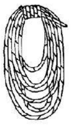 Seil	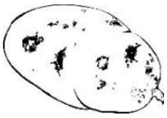 Kartoffel	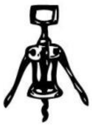 Dosenöffner
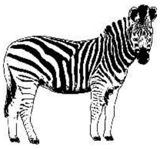 Esel	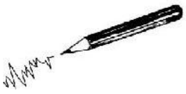 Bleistift	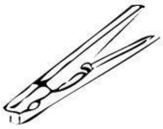 Büroklammer	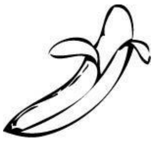 Banane	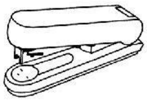 Bostitch
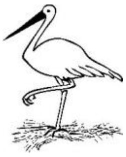 Schwan	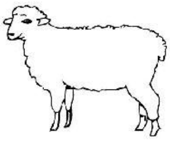 Schaf	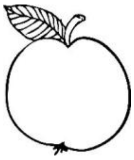 Birne	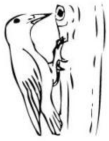 Spinne	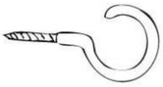 Schraube
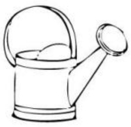 Schlauch	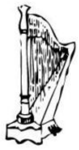 Harfe	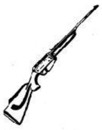 Gewehr	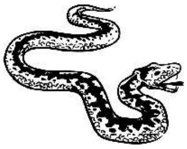 Schlange	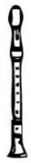 Trompete
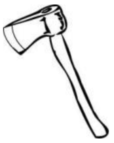 Hammer	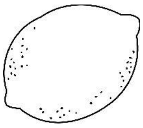 Zitrone	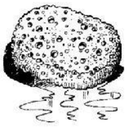 Radiergummi	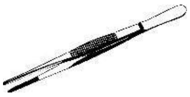 Zange	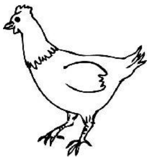 Huhn

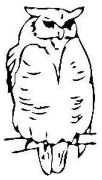	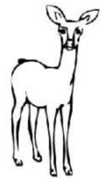	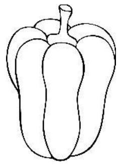	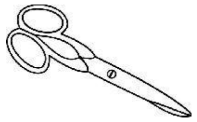	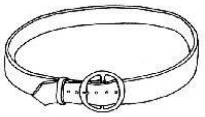
Ente	Hirsch	Peperoni	Schere	Gürtel
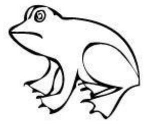	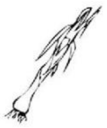	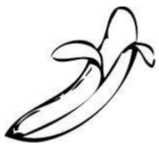	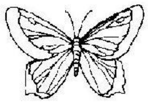	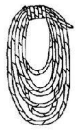
Frosch	Spargel	Orange	Libelle	Schnur
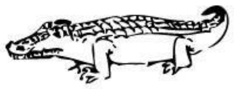	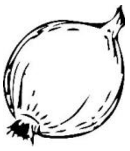	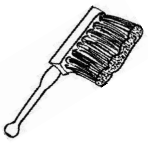	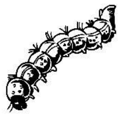	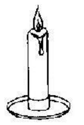
Elefant	Zwiebel	Besen	Wurm	Kerze
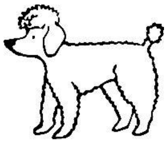	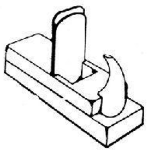	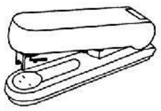	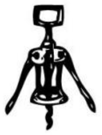	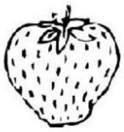
Dackel	Hobel	Locher	Korkenzieher	Erdnuss
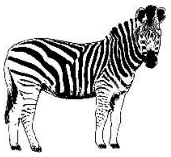	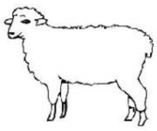	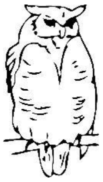	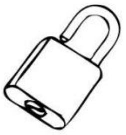	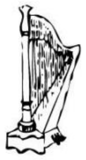
Zebra	Ziege	Eule	Schlüssel	Gitarre
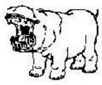	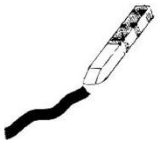	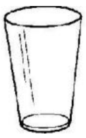	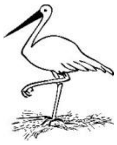	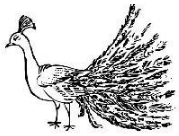
Nilpferd	Kreide	Tasse	Storch	Pfau
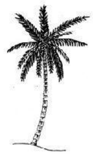	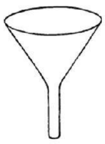	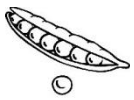	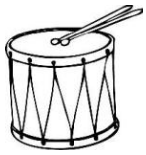	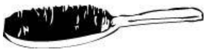
Palme	Sieb	Erbse	Trommel	Bürste

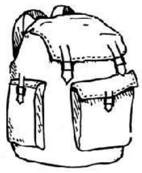
Koffer

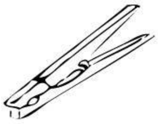
Klammer

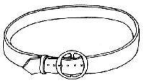
Kette

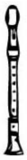
Flöte

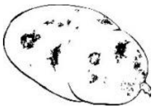
Karotte

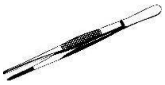
Pinzette

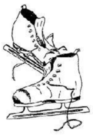
Rollschuhe

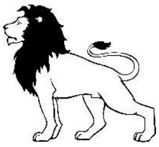
Löwe

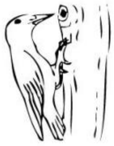
Specht

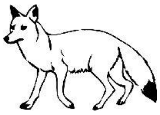
Fuchs

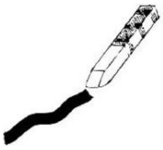
Füllfeder

Anhang D

Semantischer Entscheidungstest: Parallelversion 2

Anmerkung: Bild-Wort-Paare sind randomisiert angeordnet

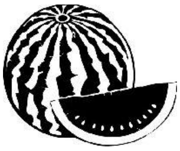	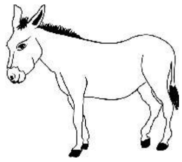	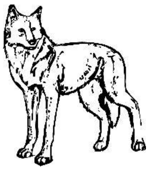	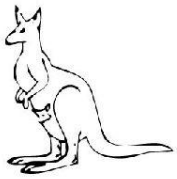	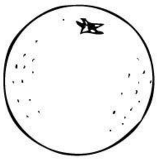
Melone	Zebra	Fuchs	Känguru	Banane
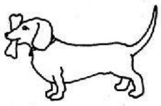	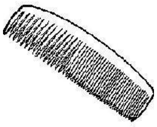	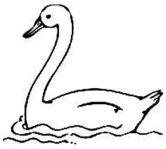	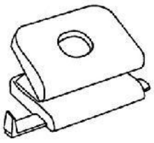	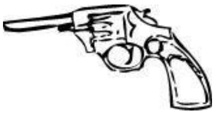
Dackel	Bürste	Schwan	Locher	Gewehr
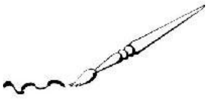	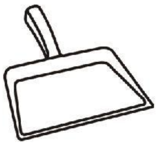	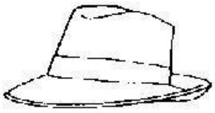	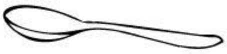	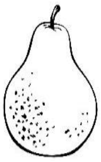
Bleistift	Besen	Hut	Gabel	Apfel
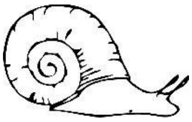	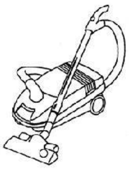	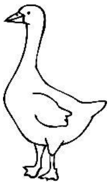	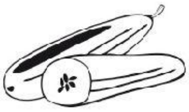	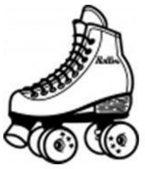
Schnecke	Staubsauger	Huhn	Erbse	Rollschuh
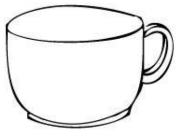	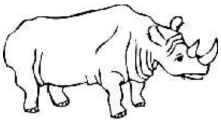	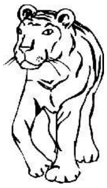	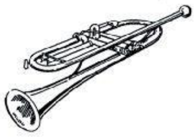	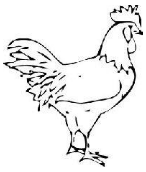
Tasse	Nilpferd	Tiger	Flöte	Hahn
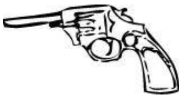	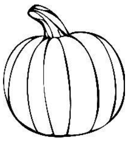	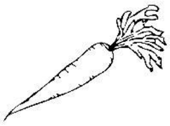	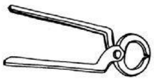	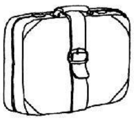
Pistole	Kürbis	Kartoffel	Pinzette	Koffer
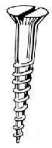	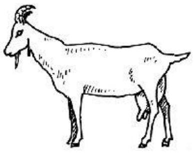	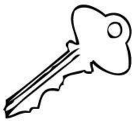	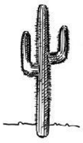	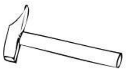
Haken	Ziege	Schloss	Palme	Beil

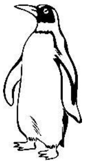	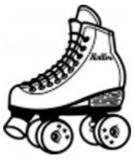	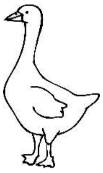	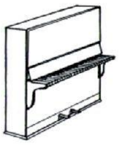	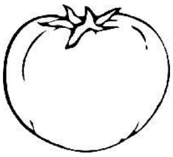
Pinguin	Schlittschuh	Gans	Trommel	Zwiebel
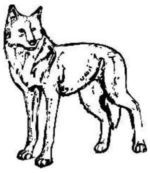	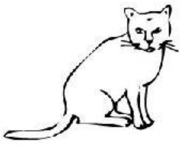	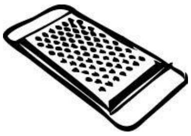	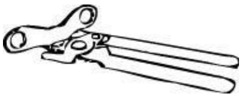	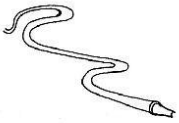
Wolf	Katze	Raffel	Dosenöffner	Giesskanne
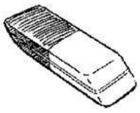	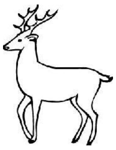	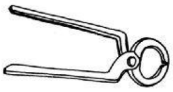	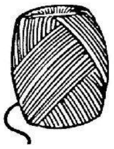	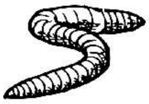
Radiergummi	Reh	Zange	Seil	Raupe
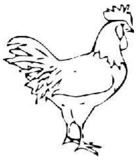	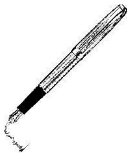	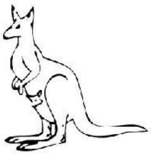	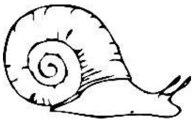	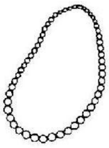
Pfau	Füllfeder	Frosch	Schlange	Gürtel
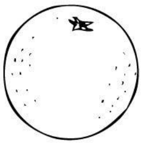	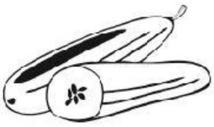	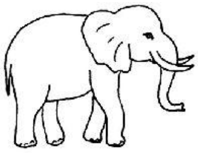	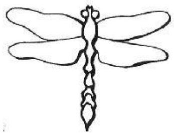	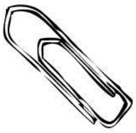
Orange	Gurke	Krokodil	Schmetterling	Klammer
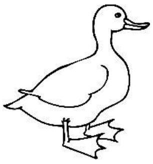	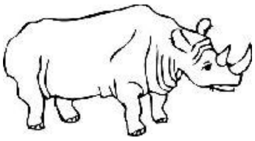	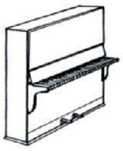	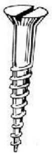	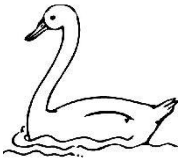
Huhn	Nashorn	Klavier	Schraube	Storch
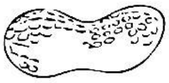	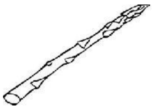	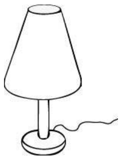	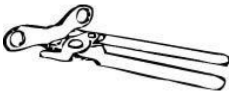	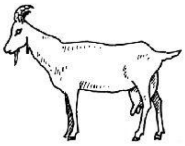
Erdbeere	Spargel	Kerze	Korkenzieher	Schaf

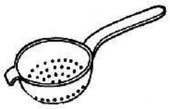
Sieb

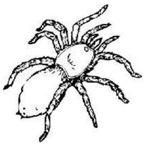
Specht

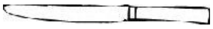
Messer

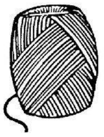
Schnur

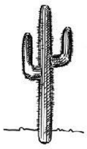
Kaktus

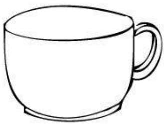
Glas

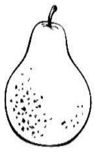
Birne

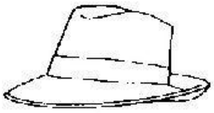
Mütze

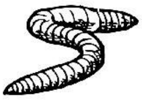
Wurm

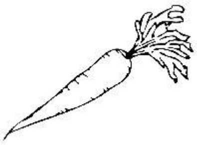
Karotte

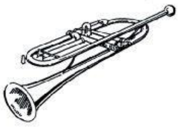
Trompete

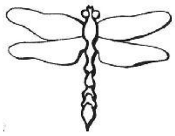
Libelle

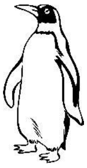
Pelikan

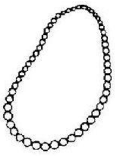
Kette

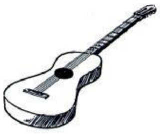
Gitarre

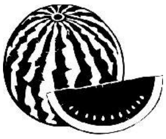
Zitrone

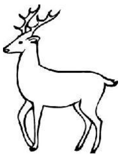
Hirsch

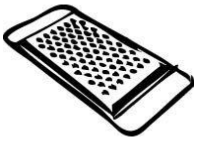
Hobel

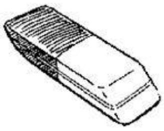
Schwamm

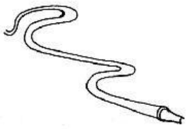
Schlauch

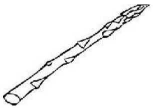
Lauch

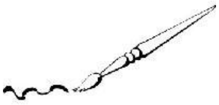
Pinsel

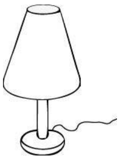
Lampe

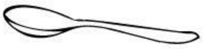
Löffel

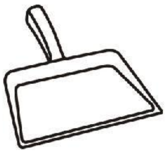
Schaufel

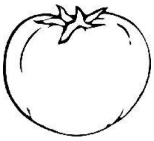
Tomate

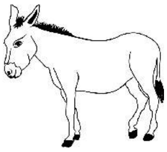
Esel

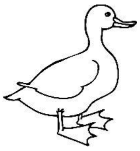
Ente

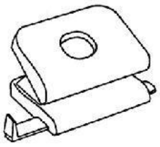
Bostitch

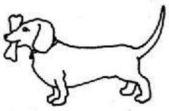
Pudel

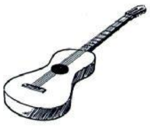
Harfe

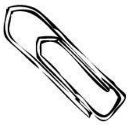
Büroklammer

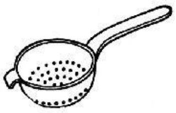
Trichter

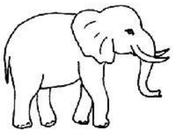
Elefant

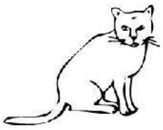
Hase

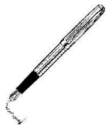
Kreide

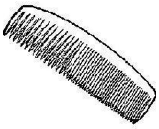
Kamm

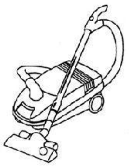
Bügeleisen

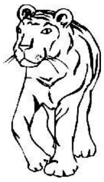
Löwe

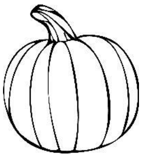
Melone

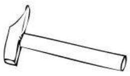
Hammer

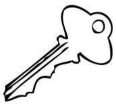
Schlüssel

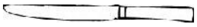
Schere

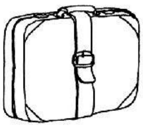
Rucksack

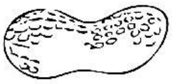
Erdnuss

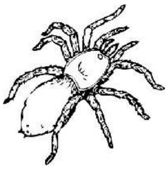
Spinne

Danksagung

Ich möchte an dieser Stelle PD Dr. Klemens Gutbrod ganz herzlich für die wertvolle Betreuung und Unterstützung bei dieser Masterarbeit danken. Weiter auch vielen Dank an Dr. Dorothea Weniger für die Zusammenstellung der verwendeten Wortpaare und für das zur Verfügung gestellte Bildmaterial. Zudem geht mein Dank an lic. phil. Rahel Schumacher für die technische Unterstützung mit dem E-Prime Programm. Vielen Dank auch all denjenigen, welche als Versuchspersonen an diesem Experiment teilgenommen haben. Auch ein grosses Dankeschön geht an Marianne, Olivia und Yvonne, welche meine Arbeit korrekturgelesen haben. Zuletzt möchte ich mich bei meinen Freunden und meiner Familie, insbesondere bei Fabian Hänggi, für die moralische Unterstützung während der Erstellung dieser Arbeit herzlich bedanken.

Selbstständigkeitserklärung zur Masterarbeit

Ich erkläre hiermit, dass ich diese Arbeit selbstständig verfasst und keine anderen als die angegebenen Quellen benutzt habe. Alle Stellen, die wörtlich oder sinngemäss aus Quellen entnommen wurden, habe ich als solche gekennzeichnet. Mir ist bekannt, dass andernfalls der Senat gemäss Artikel 36 Absatz 1 Buchstabe o des Gesetzes vom 5. September 1996 über die Universität zum Entzug des aufgrund dieser Arbeit verliehenen Titels berechtigt ist.

Ort / Datum: Basel, 21. Juni 2012

Unterschrift:

Erklärung des Einverständnisses mit der Veröffentlichung und Ausleihbarkeit der Masterarbeit

Ich erkläre hiermit, dass ich der Aufnahme der von mir verfassten Masterarbeit in den Bibliothekskatalog IDS Basel Bern sowie in die betreffende Fachbereichsbibliothek zustimme. Die Arbeit ist öffentlich zugänglich und kann von den BenutzerInnen der Bibliothek ausgeliehen werden.

Ort / Datum: Basel, 21. Juni 2012

Unterschrift:
